# Inspired by nature, refined by numbers: formal–functional bioinspiration and intelligent computation in vehicle design

**DOI:** 10.1098/rsos.241742

**Published:** 2025-05-14

**Authors:** Pooya Sareh

**Affiliations:** ^1^ School of Design, Royal College of Art, London, UK; ^2^ Creative Design Engineering Lab (Cdel), School of Engineering, Newcastle University, Newcastle upon Tyne, UK; ^3^ Higher Technical School of Engineering and Industrial Design, Polytechnic University of Madrid, Madrid, Spain

**Keywords:** vehicle design, bioinspiration and biomimetics, aerodynamic design optimization, automotive styling, artificial intelligence, integrated design process

## Abstract

For centuries, naturalist philosophers and scientists have studied the form and function of living organisms, striving to propose theories that describe the interplay between these two essential components of biological entities. This historically significant scientific quest has also emerged as a fundamental question in the design of man-made systems. In particular, humanity’s long-standing ambition to create machines and structures that imitate living organisms has driven the development of the interdisciplinary field of biomimetics. In this work, we explore and formalize various avenues for bioinspiration in engineering and industrial design, aiming to classify different types of formal and functional bioinspiration during early-stage design processes. Furthermore, we critically evaluate the evolution of the form–function relationship in vehicle design from the early twentieth century to the present era, with a particular focus on automobiles. This effort to envision the future culminates in the introduction of a framework proposed as the fifth historical phase of automobile design, where intelligent computation plays a pivotal role in integrating styling and engineering into a unified design environment. We anticipate this work to serve as a starting point for the formalization of the role of artificial intelligence in shaping the future of the design industry.

## 1. Introduction

**Figure 1.**
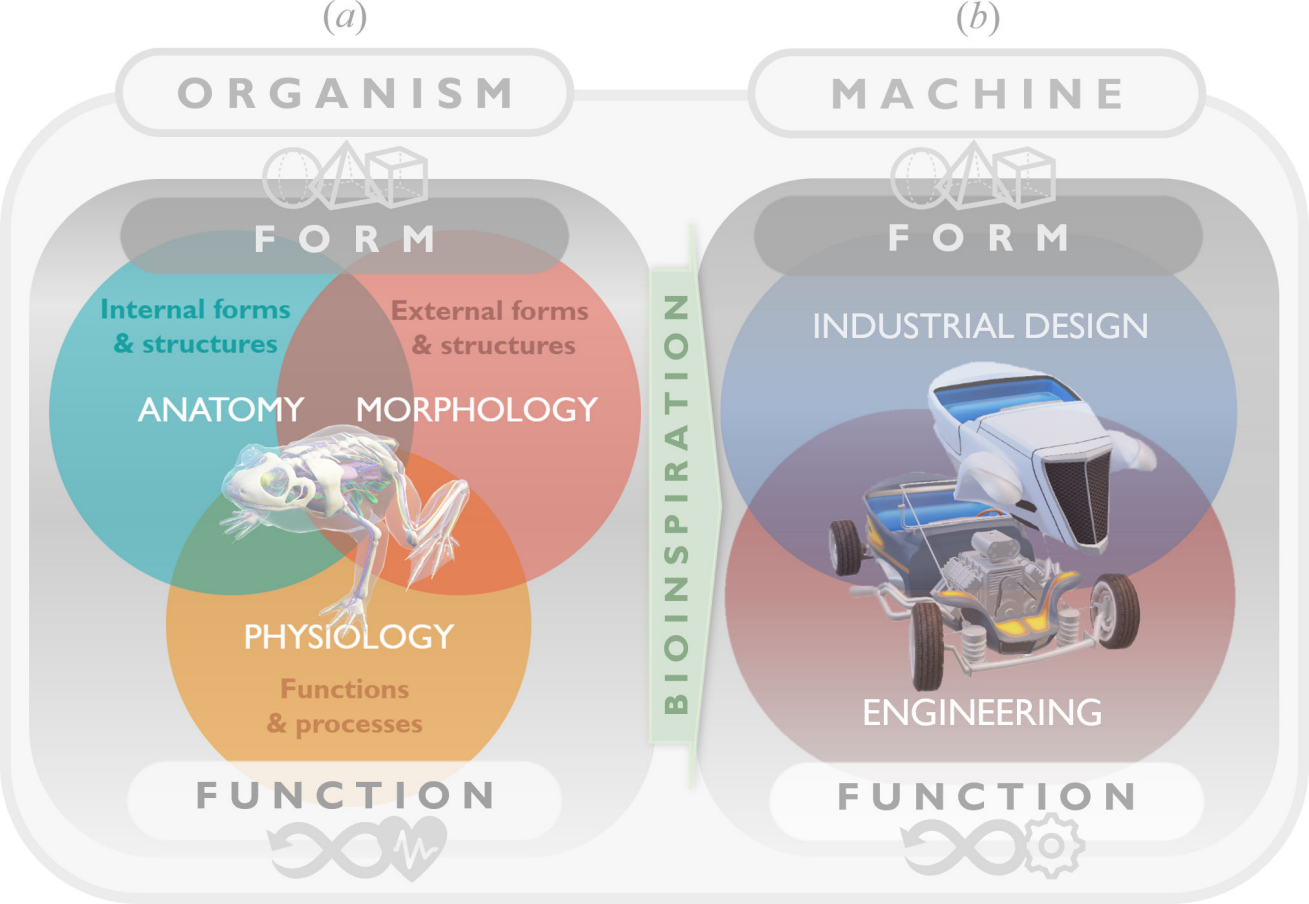
A comparison between a typical organism (the frog) and a typical machine (the automobile). (*a*) Illustration of the three disciplines that study and analyse the forms and functions associated with the frog. (*b*) Illustration of the two disciplines that examine the forms and functions associated with the automobile.

Since at least the sixth century BC [1,2], naturalist philosophers and scientists have studied the *form* and *function* of living organisms, seeking to propose theories that describe the correlations and interactions between these two essential components of biological entities [3–5]. Such endeavours have led to the development of several branches of biology which deal with different aspects of living organisms, including (i) *morphology*, the study of their *external form and structure*; (ii) *anatomy*, the study of their *internal form and structure* [6]; and (iii) *physiology*, the study of their *functions* [7] or the *processes* that occur within them [8]. Within the context of these three disciplines (see figure 1*a*), numerous studies have examined the relationships between form and function in evolutionary biology (e.g. [9–12]). It is important to note that in biology, *anatomy* and *physiology* are often considered complementary and inseparable, frequently described as the ‘*unity of form and function*’ [13,14].

This significant scientific quest has analogously appeared as a fundamental inquiry in the design of man-made objects, structures and machines since ancient times [15–19], resulting in various findings and innovations across engineering and industrial design (see figure 1*b*). Inspired by this fundamental yet eminently practical pursuit, this paper aims to examine the evolution of form–function relationships in automotive design and its influence on the development of design methodologies. Specifically, we explore and critically evaluate the evolution of the exterior design of automobiles from the early twentieth century to the present. This study will culminate in a conceptual design framework based on the historical timeline of key milestones in automotive design, offering implications for future research and practice.

The remainder of this paper is organized as follows: §2 describes and classifies various bioinspired design approaches based on different categories of biological systems and organisms, followed by a discussion of biomorphic pareidolia in design. Section 3 addresses a fundamental limitation of bioinspiration in ground vehicle design. In §4, we explore the interplay between form and function in vehicle design, with a focus on streamlining as an established design principle. Section 5 examines the evolution of automobile design methodologies, including an introduction to both legacy and current paradigms, followed by a vision for the future of automotive design in the era of artificial intelligence (AI). Finally, §6 presents the conclusions of this study.

## 2. Formal and functional bioinspiration and pareidolia

### 2.1. Bioinspiration, biomimetics and biomorphism

*Bioinspiration* is a broad term referring to the use of concepts or principles from nature to foster innovation, such as creating man-made objects or solving challenges in various fields of design, engineering and robotics. More specifically, it is defined as the ‘act of using biological phenomena to stimulate research in non-biological fields of science and technology’ [20].

The long-standing human ambition to create machines and structures that imitate living organisms has led to the development of an interdisciplinary field known as *biomimetics* [21–24] or *biomimicry* [25,26], which has opened up new avenues in engineering and industrial design (see figure 1*a,b*). Importantly, findings in these fields have significantly contributed to new insights and discussions about form–function relationships in vehicle design and mobile robotics [27–34].

*Biomorphism* is defined as ‘*the attribution of the qualities of living organisms to inanimate things*’ [35]. In the context of visual arts, biomorphism refers to ‘*abstract forms derived from or suggesting biological organisms*’, as well as the ‘*style or movement*’ characterized by such abstract forms [35]. In other words, biomorphic forms are ‘abstract’, yet they still allude to or evoke living entities, like plants and the human body [36]. The origins of the movement can be linked to painting and sculpture from 1915 to 1917, especially in the works of Jean Arp. By the 1930s, two-dimensional biomorphic shapes became prevalent in the paintings of René Magritte, Salvador Dali, Joan Miró and Fernand Léger [37].

**Figure 2.**
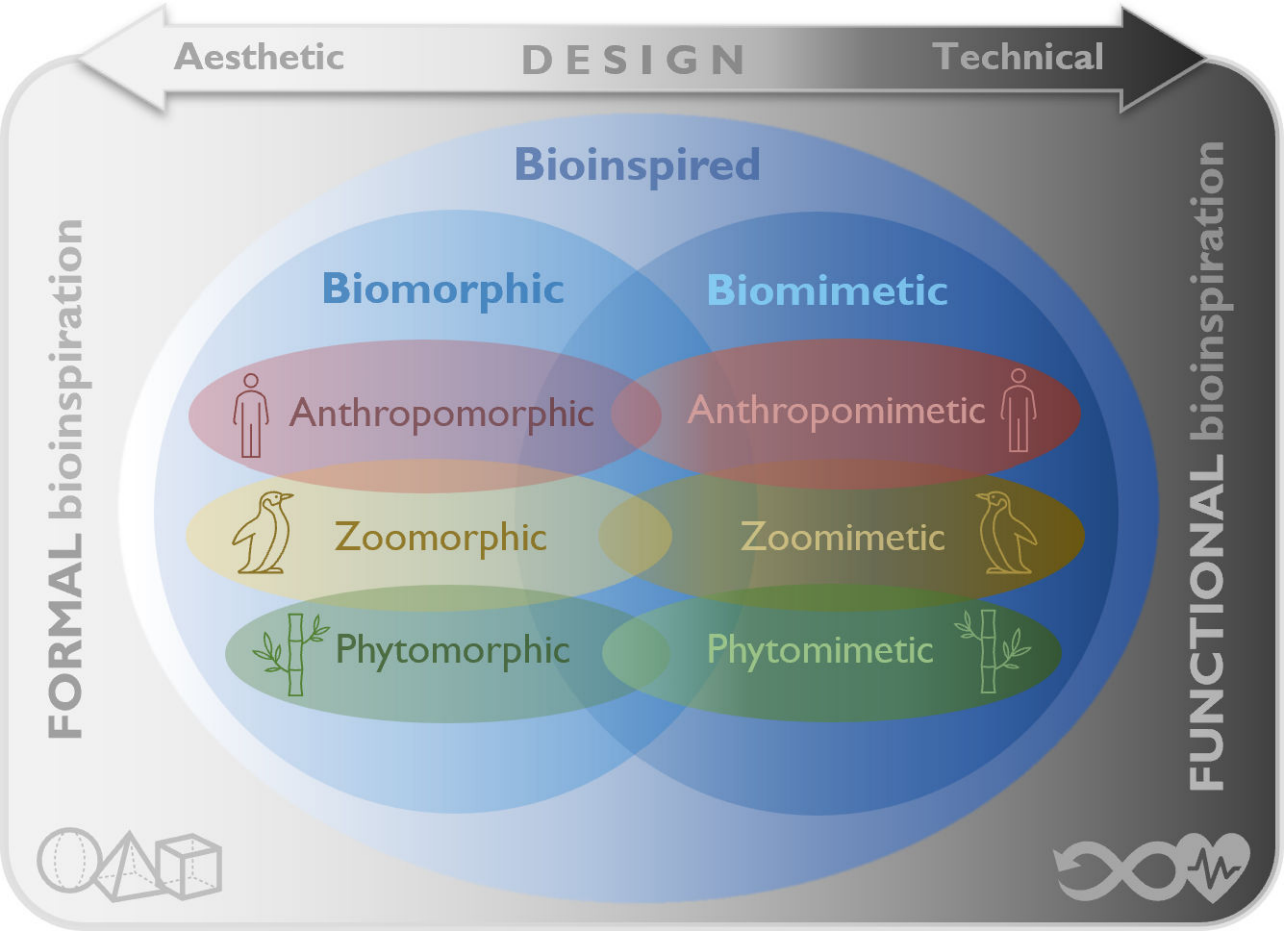
Various bioinspired design approaches based on different categories of biological systems and organisms.

Figure 2 presents a diagram illustrating various bioinspired design approaches, including biomorphic and biomimetic design, along with their respective sub-branches. As shown in this figure, biomorphic design can be categorized as *anthropomorphic*, *zoomorphic* or *phytomorphic*. Similarly, biomimetic design can be *anthropomimetic*, *zoomimetic* or *phytomimetic*. In this terminology, the Greek-origin prefixes ‘anthro-’, ‘zoo-’ and ‘phyto-’ refer to humans, animals and plants, respectively. This figure also highlights how these concepts may overlap, reflecting the relationship between formal and functional bioinspiration with aesthetic and technical design, respectively.

**Figure 3.**
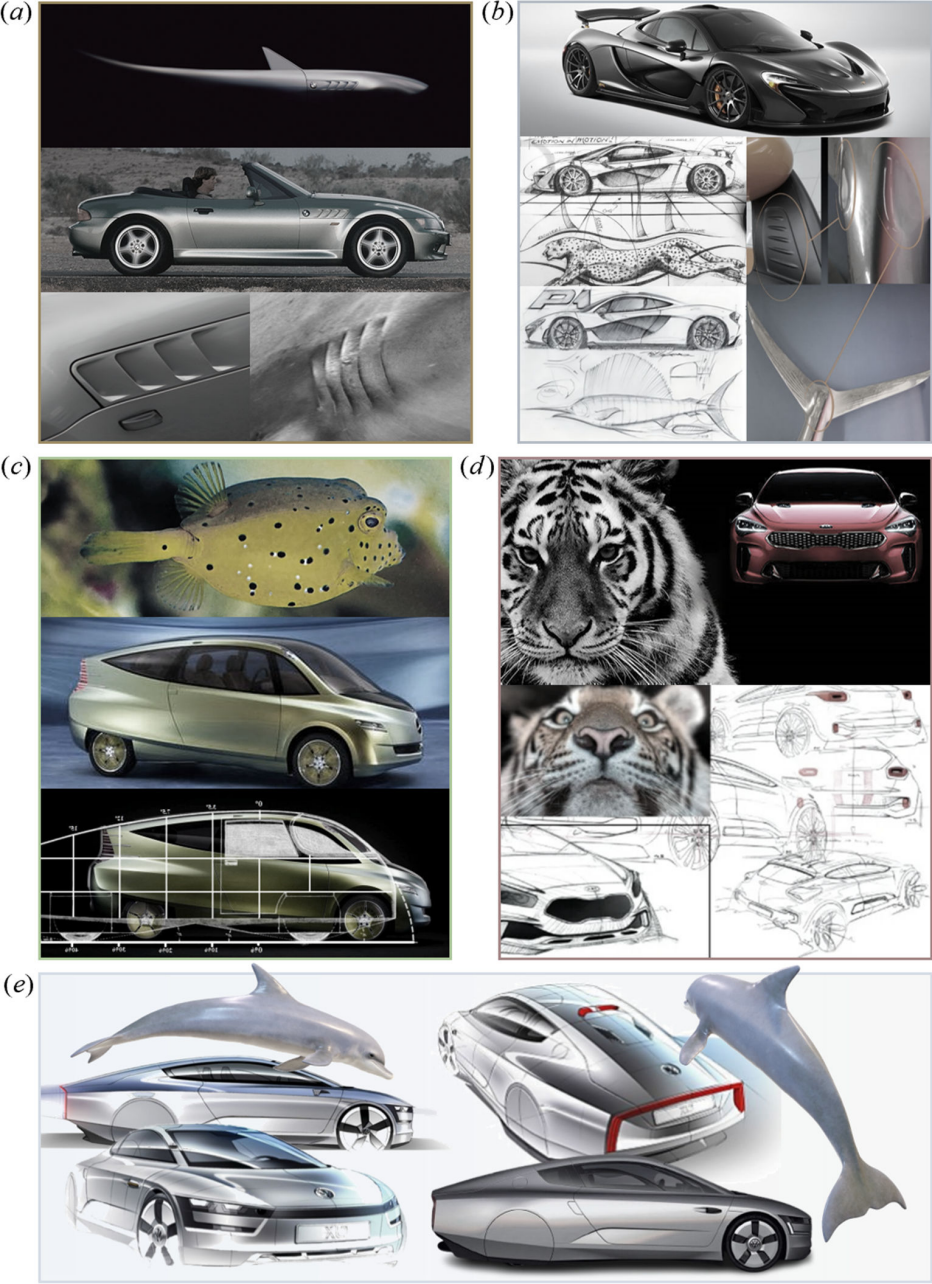
Formal and functional bioinspired features integrated into automobiles. (*a*) BMW Z3 with a shark’s fins and gills (adapted from [38]). (*b*) McLaren P1 inspired by the sailfish’s skin [39] and the cheetah (sketches by Frank Stephenson [40]). (*c*) Mercedes-Benz bionic car inspired by the yellow boxfish (adapted from [41–47]). (*d*) Kia’s tiger nose grille (adapted from [48,49]). (*e*) Volkswagen XL1 with a shape reminiscent of a dolphin (adapted from [50]).

In automotive design, animal names and forms are often employed to symbolize a wide range of desirable qualities, such as speed, agility, power and strength. Notably, the emotional connection evoked by this approach serves as an effective marketing strategy. Figure 3 presents various examples of bioinspired features, both formal and functional, integrated into automobiles. Figure 3*a* illustrates a BMW Z3 with a shark’s fins and gills [38], with the features used in the car’s publicity campaigns. Figure 3*b* shows a McLaren P1 inspired by the sailfish’s skin [39] and the cheetah [40]. Figure 3*c* displays a Mercedes-Benz bionic car inspired by the yellow boxfish [41–47], while figure 3*d* depicts a Kia design showcasing the distinctive tiger nose grille [48,49].

Another notable example is the Volkswagen XL1, inspired by the streamlined body of a dolphin, as shown in figure 3*e*. Scientists have demonstrated that dolphins have evolved exceptionally streamlined bodies, enabling them to reduce both form drag and friction drag [51,52]. When viewed from above, the XL1’s shape is reminiscent of a dolphin, particularly at the rear [53]. Regarded as the world’s most aerodynamically efficient car of its time, with a drag coefficient of 0.189 [54,55], the XL1’s body was meticulously optimized for aerodynamic performance. It is widest at the front and gradually tapers towards the rear, with body lines precisely shaped to align with the airflow, thereby minimizing aerodynamic drag [53]. This represents an excellent example of *formal–functional bioinspiration*, where zoomorphic styling and zoomimetic engineering optimization have resulted in an appealing, highly efficient automobile.

**Figure 4.**
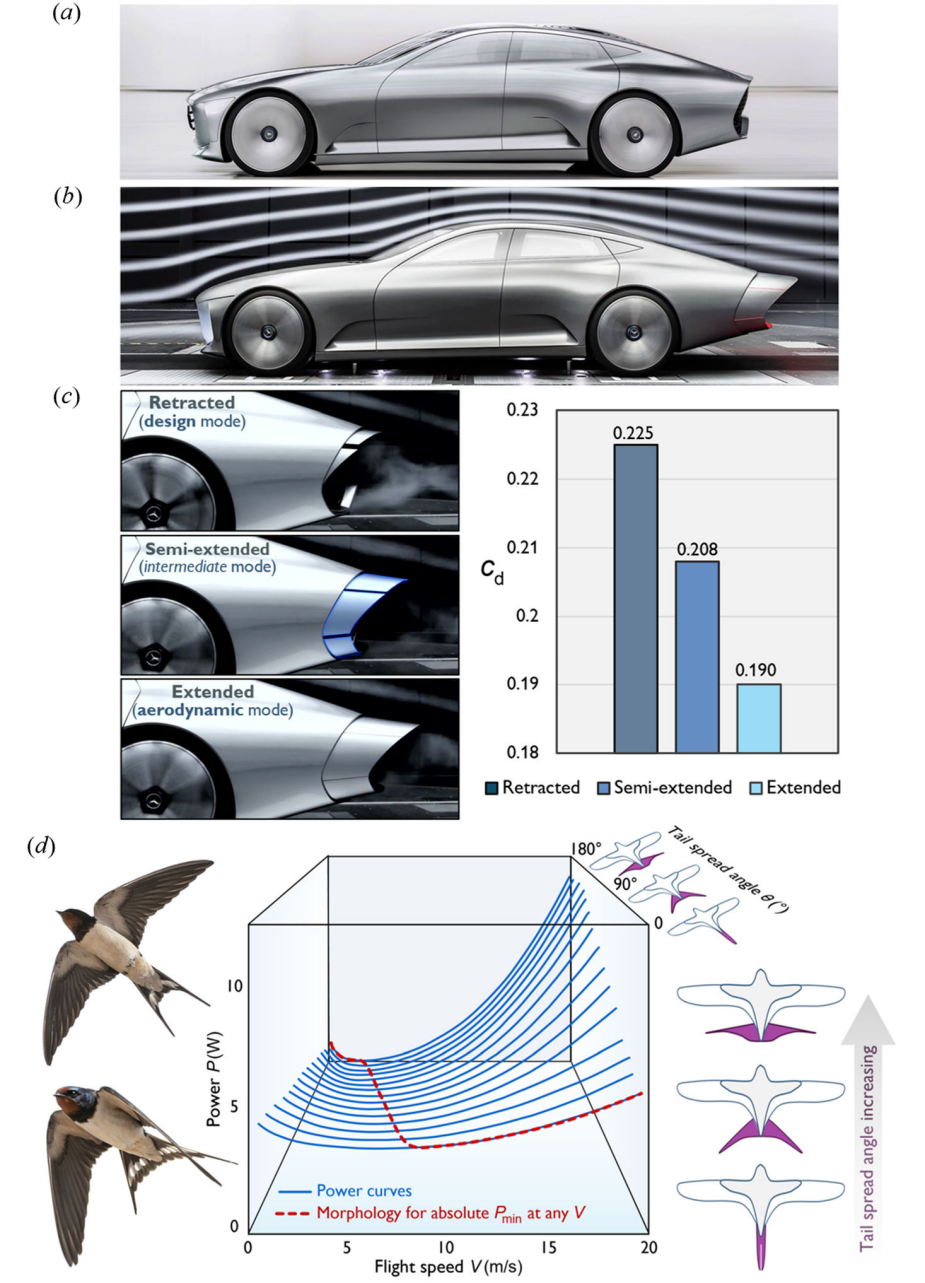
Tail reconfiguration as a strategy for enhancing aerodynamic efficiency in both vehicle design and nature. (*a–c*) The Mercedes-Benz Concept IAA (Intelligent Aerodynamic Automobile), featuring an adaptive tail design for drag minimization (adapted from [56,57]). (*d*) Left: a typical barn swallow in two different flight modes. Right: the influence of the swallow’s tail configuration on the power required for flight (adapted from [58]).

The Mercedes-Benz Concept IAA (Intelligent Aerodynamic Automobile), shown in figure 4*a,b*, features active aerodynamic elements—including front flaps, a louvre, active rims and a rear extension—that adjust to optimize airflow and reduce drag at higher speeds. At 80 km h^−1^, the car automatically transitions from ‘design mode’ to ‘aerodynamic mode’, triggering a series of active aerodynamic adjustments that alter its appearance [56].

While not explicitly described as bioinspired, this design is reminiscent of how animals, such as birds and fish, adjust their body shapes to enhance aerodynamics or hydrodynamics. Active car body elements such as spoilers, diffusers and wheel housings that extend and retract mimic these natural adaptations. Notably, the Concept IAA’s shape-shifting tail design reduces its drag coefficient from 0.225 to 0.19 (see figure 4*c*) [57]. This is analogous to the biological adjustments of birds’ tails, such as those of swallows and Harris hawks, where structural reconfigurations minimize drag and improve flight efficiency.

As illustrated in figure 4*d*, swallows modify their tail shape to regulate the power required for flight. At slower speeds, the tail reduces the workload on the wings, thereby lowering the power needed. At higher speeds, the tail can provide additional lift, such as during a turn, though this comes at the cost of increased drag. The least amount of power required at any speed is achieved by adopting the tail morphology represented by the dashed line [58].

Similarly, the role of the Harris hawk’s tail in stabilizing and balancing flight has been demonstrated through experiments with a trained Harris hawk in a tilting wind tunnel. At higher glide speeds, the bird adopts a posture with its wings flexed and swept slightly backward, while its tail remains furled to minimize drag. Conversely, at lower speeds, the hawk extends its wings more broadly, shifting the centre of lift forward, ahead of its centre of gravity. This shift increases the risk of the bird pitching backward at low speeds. To maintain stability, the hawk uses its tail to generate additional lift, preventing a loss of balance [58,59].

**Figure 5.**
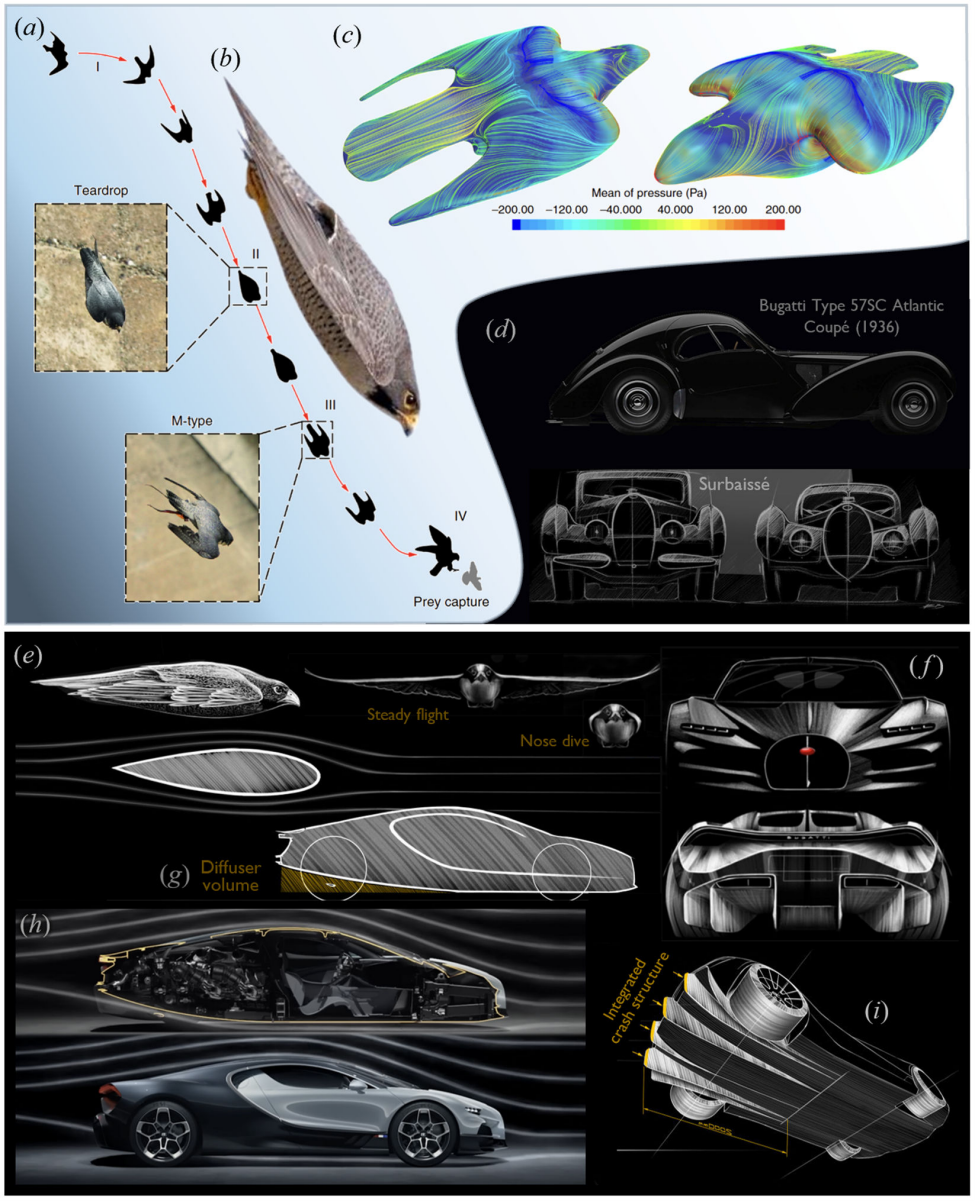
Formal–functional bioinspiration from the peregrine falcon in the early-stage design of the Bugatti Tourbillon. (*a*) Flight path of a peregrine falcon in a stoop, illustrating the teardrop shape and the M shape (adapted from [63]). (*b*) Profile of the falcon in its most aerodynamically efficient body configuration (adapted from [64]). (*c*) Near-surface streamline pattern of airflow and mean-pressure contour lines over the falcon (adapted from [63]). (*d*) Bugatti Type 57SC Atlantic Coupé (1936), a well-known example of ‘Surbaissé’ cars (adapted from [65,66]). (*e*) The highly streamlined profile of the peregrine falcon in two different configurations. (*f–i*) Various features and design elements of the Bugatti Tourbillon (adapted from [65]). (*f*) Front and rear designs. (*g*) Diffuser volume. (*h*) Streamlined profile and internal compartments. (*i*) Integrated crash structure.

Another well-studied shape-shifting bird of prey is the peregrine falcon (*Falco peregrinus*), which holds the title of the fastest bird in the world, capable of reaching diving speeds of up to 320 km h^−1^. During its dive, the falcon adjusts the shape of its wings, gradually pulling them closer to its body as it accelerates. At maximum speed, the falcon folds its wings tightly against its elongated body, creating a ‘vacuum pack’ effect to minimize air resistance [60–62]. Figure 5*a* shows the flight path of a peregrine falcon in a stoop while in the teardrop shape and the M shape. Figure 5*b* provides a close-up of a typical example of the bird in its most aerodynamically efficient body configuration, while figure 5*c* illustrates the near-surface streamline pattern of airflow and the mean-pressure contour lines over the falcon.

The Bugatti Tourbillon (figure 5*e*–*i*) is another case study with well-documented notes on sources of inspiration in its creation. Based on Bugatti’s official conceptual design presentations [65], achieving extremely efficient aerodynamic design was particularly prioritized during the conceptual design phase, drawing on two primary sources of inspiration.

First, the historical achievements of Bugatti in the 1930s involved the creation of low-slung chassis, leading to ‘Surbaissé’ (meaning ‘lowered’) cars (figure 5*d*), which achieved both a sportier stance and enhanced aerodynamics. Well-known examples include the Type 57SC ‘Aérolithe’ concept and the later Atlantic production models (the ‘S’ stood for ‘Surbaissé’ and the ‘C’ for ‘Compresseur’ (Compressor)). Inspired by this legacy, Frank Heyl, Bugatti’s Director of Design, explained: ‘*If the car is lower, it looks wider and the size of the wheels are emphasized; it looks like there is tension in the muscles, a posture ready to pounce. Every design decision is geared towards creating a sense of speed even at a standstill’* [65]. This vision led to minimizing the frontal area, lowering the roofline and lowering the driver’s position (figure 5*f*).

Second, the highly streamlined profile of the Bugatti Tourbillon is said to be inspired by the peregrine falcon [65], as depicted in figure 5*e*–*h*. Specifically, the falcon’s fully folded body configuration during dives, which minimizes frontal area and aerodynamic drag, influenced the overall architecture of the Tourbillon. This serves as an example of formal–functional bioinspiration in early-stage vehicle design.

Other innovative features of the Bugatti Tourbillon include a diffuser design for aerodynamic stability, which begins to slope upward just behind the passenger cabin (figure 5*g*). The diffuser incorporates a new crash concept fully integrated into its design, making it highly effective yet concealed from sight, thus enabling the open rear-end design (figure 5*i*). Notably, this mid-engine hybrid sports car can achieve its estimated top speed of 445 km h^−1^ without the need for a raised rear wing.

**Figure 6.**
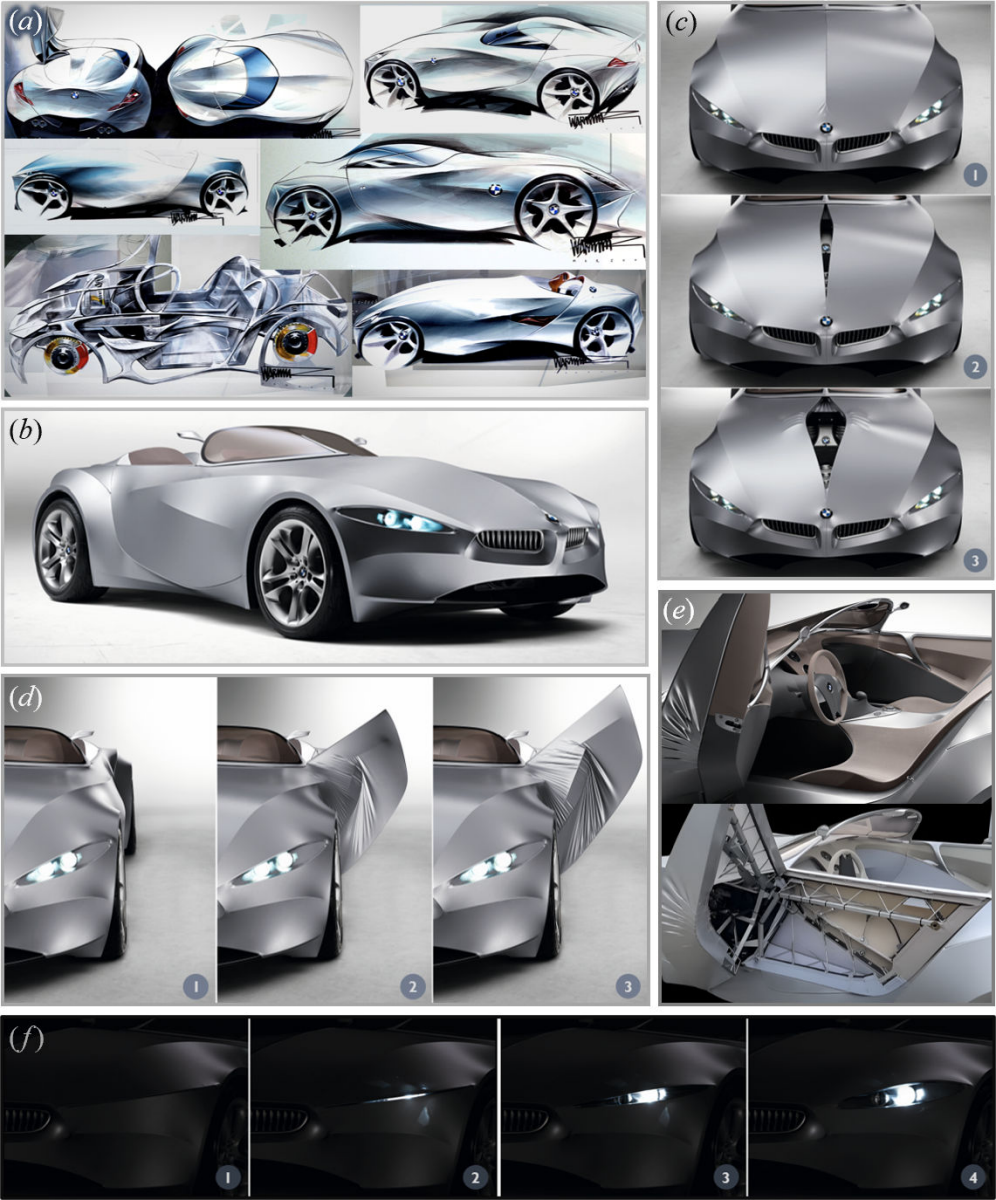
The GINA Light Visionary Model by BMW. (*a*) Anders Warming’s sketches (adapted from [69]). (*b*) Three-quarter view. (*c*) Morphing bonnet in three different states. (*d*) Morphing door in three different states. (*e*) External and internal structures of the morphing door. (*f*) Adaptive headlight in four different states (adapted from [67]).

BMW introduced a shape-shifting, fabric-skinned sports car concept called the GINA Light Visionary Model [67], shown in figure 6, in 2008. GINA, standing for ‘Geometry and functions In ‘N’ Adaptions’, featured an actively flexible, elastic skin technology that allowed the vehicle body to adapt its shape according to different driving and environmental conditions.

The overall design philosophy of GINA draws inspiration from living organisms that morph continuously to adapt to their environment. While not explicitly described as bioinspired, the highly organic concept incorporates several features reminiscent of biological systems and structures. Chris Bangle, BMW’s head of design at the time, stated [68]: ‘*Emotion is really the added value to this. I mean, one way is saying you get better function or better alternative ways of doing things, but we really want to achieve a higher emotional plane out of this. And finally, I think this is, to me, one of the most important things: the level of humanistic content that we can bring with GINA. GINA should be about the human in the loop—the human way of doing things.’* Moreover, Anders Warming, GINA’s exterior designer, mentioned [69]: ‘*[The flexible skin] allowed us to make a car that has almost human traits, human expressions... We have the opening for the central part of the bonnet, and I think, most prominently, the door openings—a door without a front door cut; so when it opens, you get these folds, that basically are given by nature*.’

From these comments, we can see that the design of GINA subtly incorporates bioinspiration by mimicking natural forms and human-like expressions. Furthermore, Warming’s sketches of the concept’s exterior, shown in figure 6*a*, while not referring to any specific biological entity, exhibit a strong sense of organic fluidity.

The first remarkable feature of GINA is an actively flexible, synthetic, water-resistant fabric skin that replaces the rigid body panels found in conventional cars. This flexible covering functions like biological membranes and tissues—such as skin or muscle—allowing the car’s exterior to adjust dynamically. The transformation is facilitated by an underlying shape-shifting substructure composed of an aluminium wire frame with carbon-fibre struts [70], powered by hydraulic-electric actuators. This system allows the vehicle to change its form based on driving conditions, either automatically or through driver input. The structure–membrane combination is reminiscent of biological musculoskeletal systems, where bones act as a substructure enabling controlled body movements.

One notable aspect of GINA’s design is its minimalist approach to the outer skin, which can be seen as an attempt to mimic the seamlessness of biological skin in animals and humans. The vehicle’s body consists of only four components. The largest component runs from the front of the vehicle to the edge of the windscreen and extends along the sides to the rear edges of the doors. The two elongated side panels start at the front where the rocker panels emerge and continue over the rear wheel arches, merging into the rear. Finally, the assembly is completed by adding the central rear deck as the fourth component.

Among the various morphing parts in GINA’s exterior design, perhaps the most striking feature is its adaptive headlights, which are inspired by—or at least reminiscent of—human or animal eyelids (figure 6*f*). This design mimics the protective function (guarding against external debris and damage) and light-exposure regulation of biological eyelids, with the obvious distinction that headlights are light sources rather than receivers. Consequently, this feature serves as an excellent example of formal–functional bioinspiration in vehicle design.

However, the scope of bioinspiration in the other components of the concept remains primarily at a formal level, as features like the morphing bonnet slit (figure 6*b*,*c*) and biomorphic ‘bird wing’ style doors (figure 6*d,e*) are not specifically designed to achieve engineering performance metrics, such as minimizing aerodynamic drag. Nevertheless, the bioinspired design vision introduced with GINA could potentially be applied to future concept vehicles, where body components adapt their shapes to meet targeted functional requirements.

**Figure 7.**
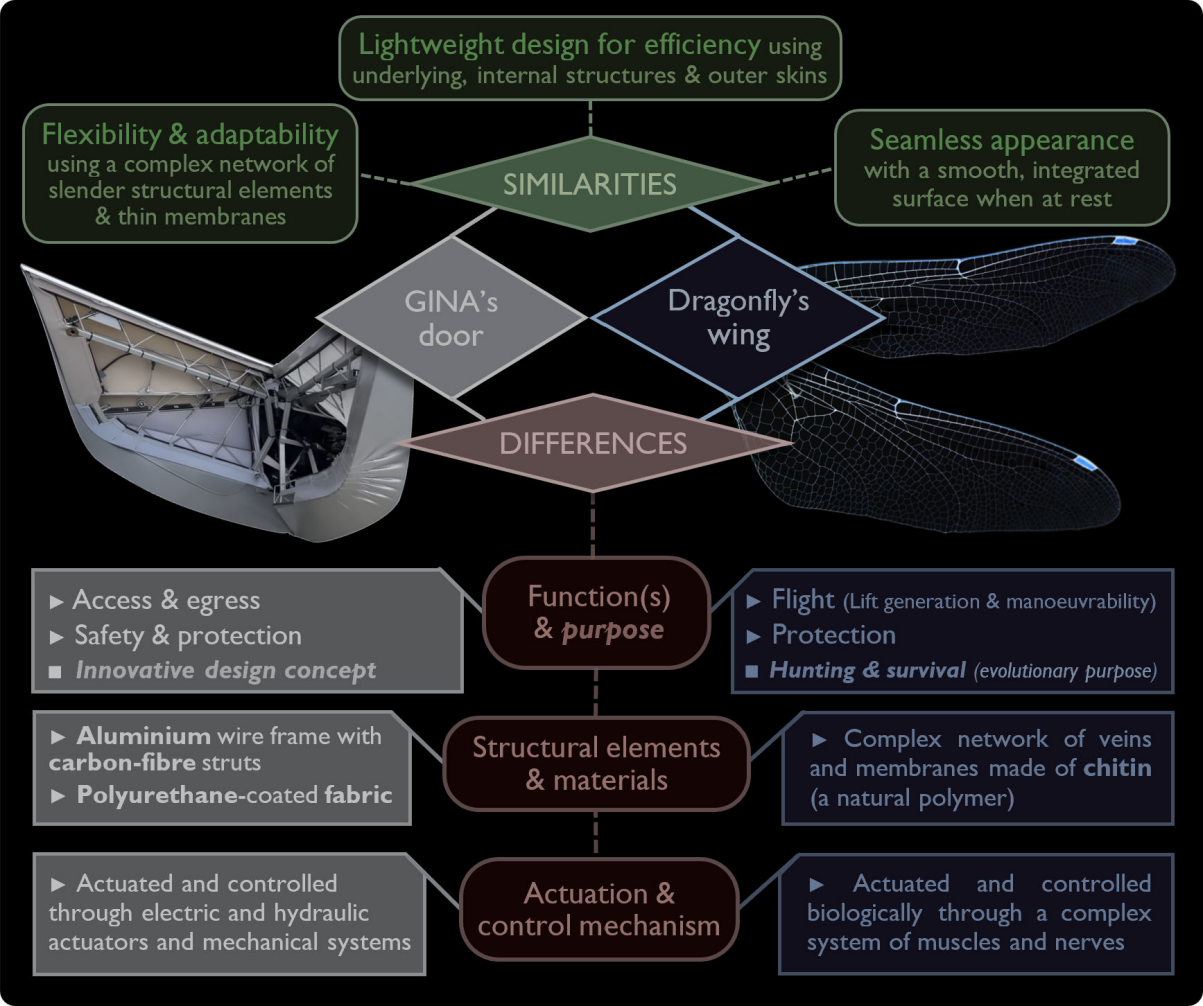
A comparative analysis of the GINA concept car’s morphing door and a dragonfly’s wing, highlighting their similarities and differences.

The internal structure of GINA’s door can be compared with the anatomy of insect wings, such as those of a dragonfly. Figure 7 illustrates this comparison, highlighting both similarities and differences in their design and functionality. Notably, both structures share key characteristics, including lightweight efficiency, flexibility, adaptability and a seamless appearance. The GINA door and the dragonfly wing utilize a complex network of slender structural elements and thin membranes, ensuring both structural integrity and adaptability. When at rest, they exhibit a smooth, integrated surface that enhances aerodynamics and aesthetics. Despite these shared principles, they differ significantly in functions, materials and actuation mechanisms, as summarized in figure 7.

### 2.2. Bioinspired design versus biomorphic pareidolia

People may experience moments in which they attribute certain designed objects or products to unrelated features found in organisms or other objects. In psychology, *apophenia* is defined as the tendency to perceive meaningful connections between unrelated or random things, such as objects or ideas [71,72]. *Pareidolia*, a specific type of apophenia, refers to the tendency to perceive a specific, often meaningful pattern or image of something that does not exist within a random or ambiguous visual pattern [72,73].

**Figure 8.**
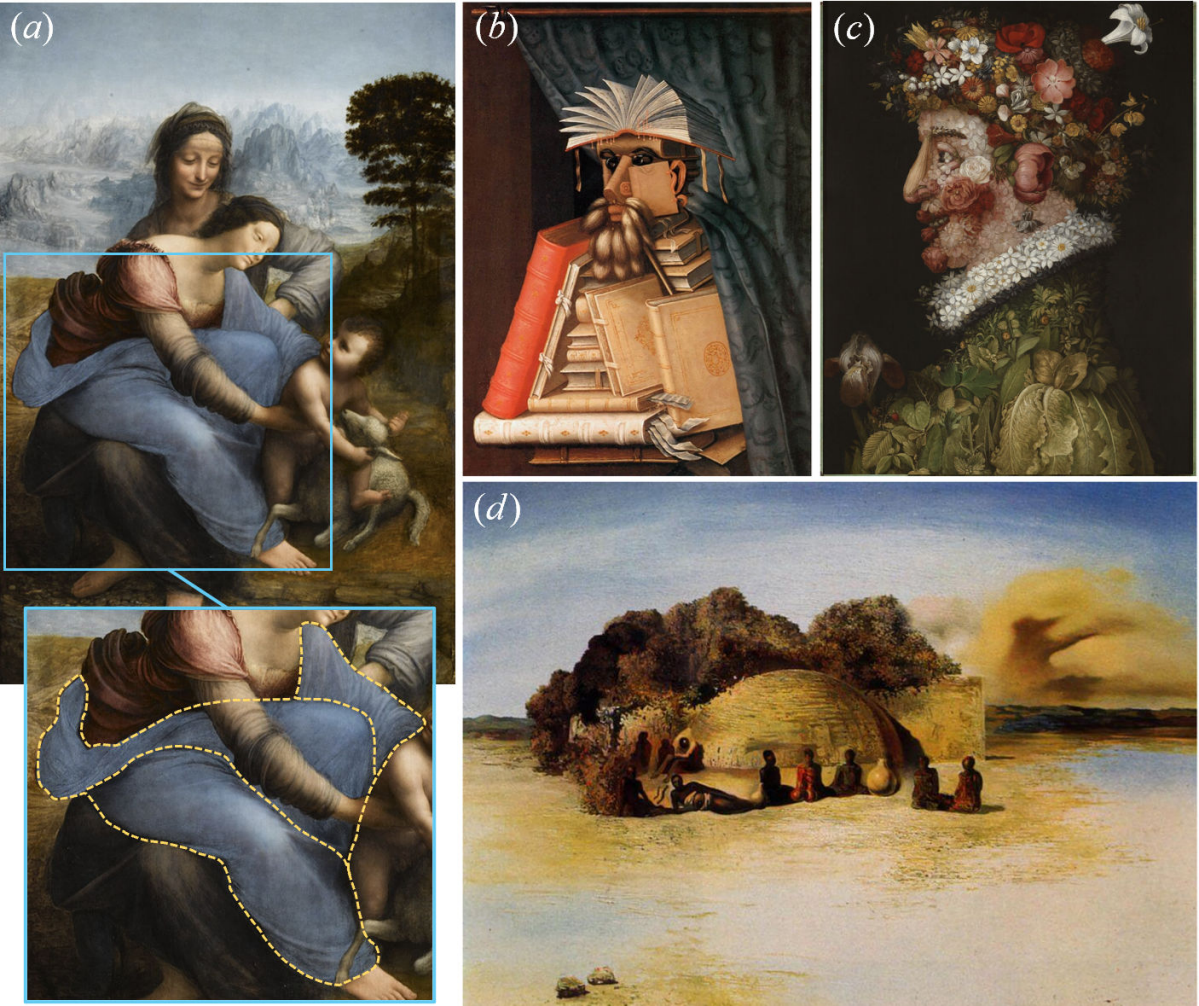
Biomorphic pareidolia in various Renaissance and twentieth-century paintings. (*a*) Zoomorphic pareidolia in *The Virgin and Child with Saint Anne* (c. 1501−1519) by Leonardo da Vinci (1452−1519). (*b–c*) Anthropomorphic pareidolia in the works of Giuseppe Arcimboldo (1527−1593): (*b*) *The Librarian* (1566), and (*c*) *The Four Seasons: Spring* (1563). (*d*) Anthropomorphic pareidolia in *Paranoiac Face* (1937) by Salvador Dali (1904−1989).

In the context of visual arts, many artists have evoked pareidolia through intentional compositions that lead viewers to perceive familiar shapes formed by seemingly unrelated visual elements. A well-known example is the zoomorphic pareidolia in *The Virgin and Child with Saint Anne* by Leonardo da Vinci (1452−1519), depicted in figure 8*a*. In this painting, the Virgin’s garment reveals the profile of a bird (kite or vulture), which Sigmund Freud used as the basis for a psychoanalytic examination of da Vinci [74]. Figure 8*b*,*c* shows two examples of anthropomorphic pareidolia in the works of another Italian Renaissance painter, Giuseppe Arcimboldo (1527−1593), renowned for his imaginative portraits composed of a wide range of elements such as animals, fruits, plants, flowers, candles and books [75]. Furthermore, anthropomorphic pareidolia is often found in the works of twentieth-century surrealist artists, such as Salvador Dali’s *Paranoiac Face* (1937), shown in figure 8*d*.

**Figure 9.**
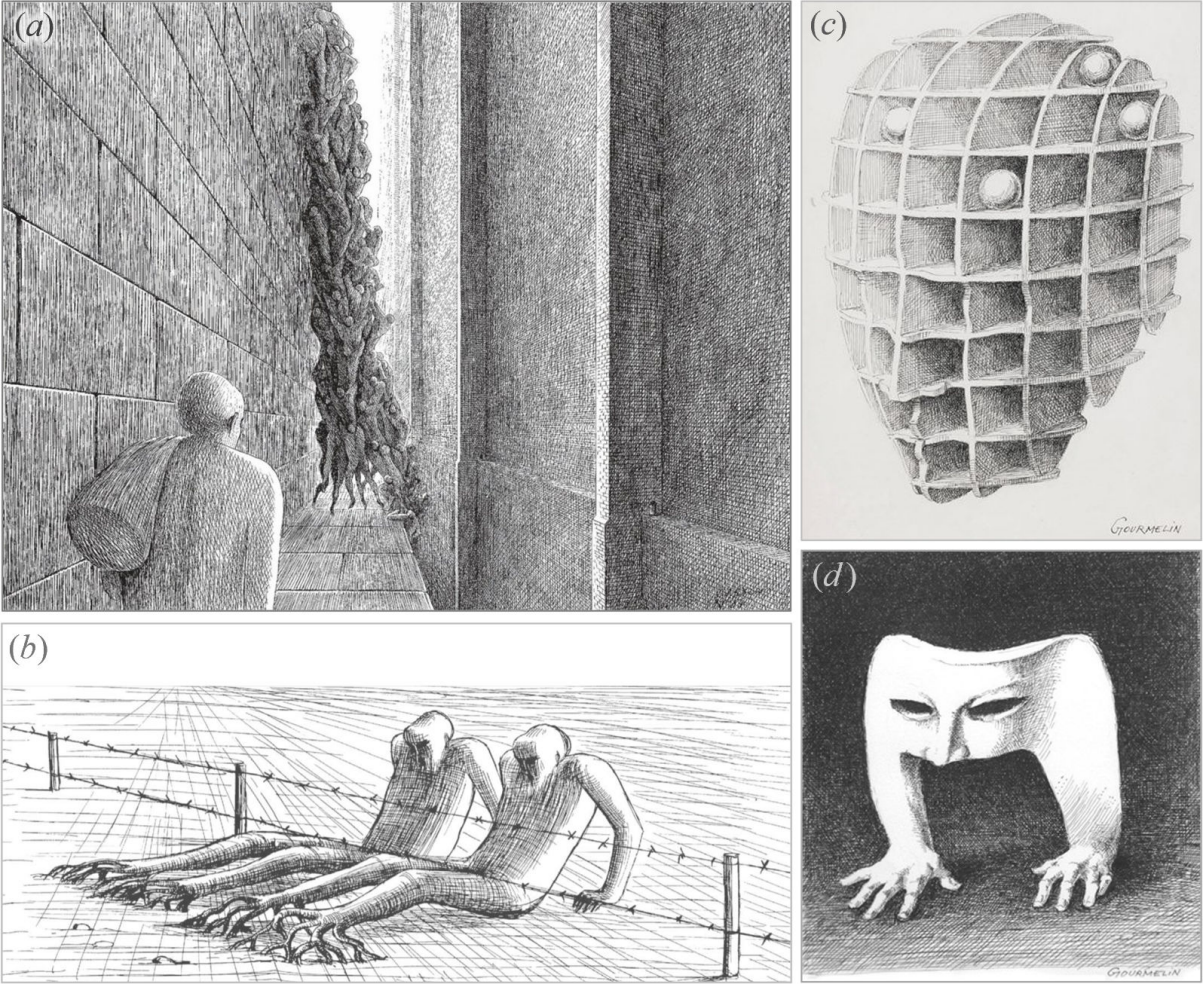
Biomorphic pareidolia in surrealist drawings by French artists of the mid-twentieth century. (*a–b*) Dystopian phytomorphic drawings by Jacques-Armand Cardon (images adapted from [76,77]). (*c–d*) Drawings featuring anthropomorphic and zoomorphic elements by Jean Gourmelin (images adapted from [78,79]).

Notably, biomorphic pareidolia was a key element in the surrealist drawings of mid-twentieth-century French cartoonists and illustrators. Examples include the dystopian phytomorphic works of Jacques-Armand Cardon (b. 1936) and the darkly humorous illustrations of Jean Gourmelin (1920−2011), which feature anthropomorphic and zoomorphic elements, as shown in figure 9.

In the work shown in figure 9*a*, Cardon depicts a narrow alleyway dominated by a towering mass of human figures, intertwined as they climb within a confined vertical space between two walls. The human bodies are intricately connected, resembling the structure of a plant with roots, with each individual figure forming part of this organic, root-like network. Figure 9*b* depicts another striking phytomorphic/anthropomorphic example by the same artist: two elongated humanoid figures attempting to cross a barbed wire fence. Their lower bodies resemble tree trunks with sprawling roots, creating a compelling fusion of human and botanical forms. It is a striking merger of human and botanical imagery, inviting viewers to explore their own interpretations of the relationship between the two.

Figure 9*c* features a drawing by Gourmelin depicting a three-dimensional grid structure that resembles a human head, combining elements of geometry and human anatomy. The intersecting planes merge geometric precision with human anatomy, producing a recognizable yet abstract representation of the human form and emphasizing its anthropomorphic essence. In another example by Gourmelin, figure 9*d* presents a hybrid anthropomorphic–zoomorphic creation, combining elements of a human face mask with human hands that appear to crawl like animal limbs. The absence of a complete body amplifies the image’s haunting and surreal atmosphere, giving the impression that the mask has come to life.

In the context of industrial and automotive design, it is important to distinguish between two terms: *X-inspired design* and *X-morphic pareidolia*. X-inspired design refers to a design that intentionally incorporates formal and/or functional elements inspired by X, where X represents the source of inspiration. In this case, the designer(s) deliberately integrate specific features of X into their design work. By contrast, X-morphic pareidolia describes a psychological phenomenon in which observers or consumers spontaneously perceive ‘X-like’ forms or features in a design, even though these elements were not intentionally included by the designer(s).

**Figure 10.**
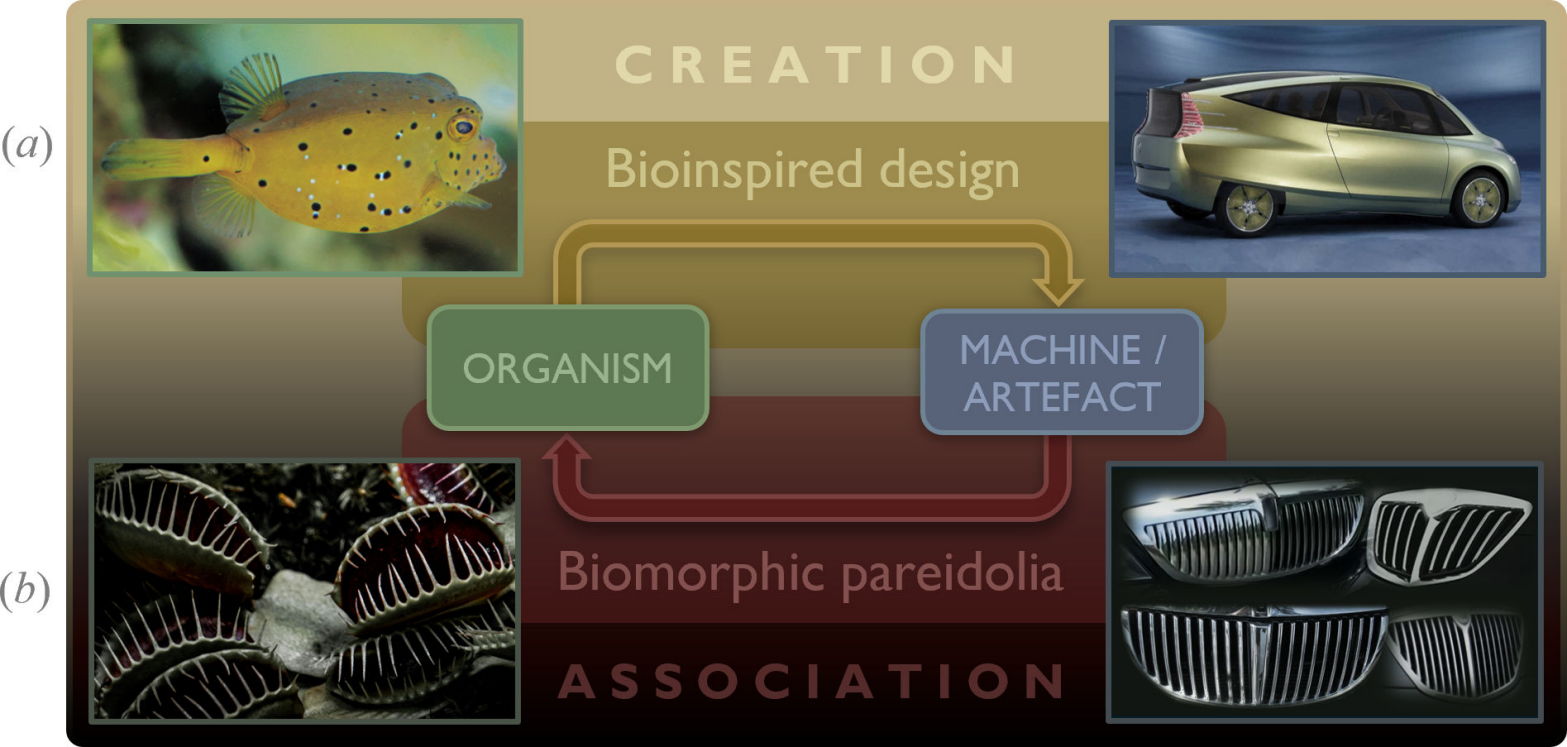
An example of bioinspired design versus biomorphic pareidolia in automotive design. (*a*) The process of bioinspiration and biomimetics, showcasing the Mercedes-Benz bionic car (right), which was inspired by the aerodynamically efficient form of the yellow boxfish (left). (*b*) The process of biomorphic pareidolia, illustrating the resemblance between the grilles of cars (right) and carnivorous plants (left).

To further elaborate on the above concepts, figure 10 provides visual examples that highlight the interplay between bioinspired design and biomorphic perception in both pre-creation and post-creation contexts. Specifically, figure 10*a* illustrates the *pre-creation* process of *bioinspired design* depicting the Mercedes-Benz bionic car (right), which was inspired by the highly aerodynamically efficient form of *Ostracion Cubicus*, commonly known as the yellow boxfish [41–46]. By contrast, figure 10*b* presents an example of a *post-creation* process called *biomorphic pareidolia*, which refers to the association between a man-made object or product and a biological organism.

**Figure 11.**
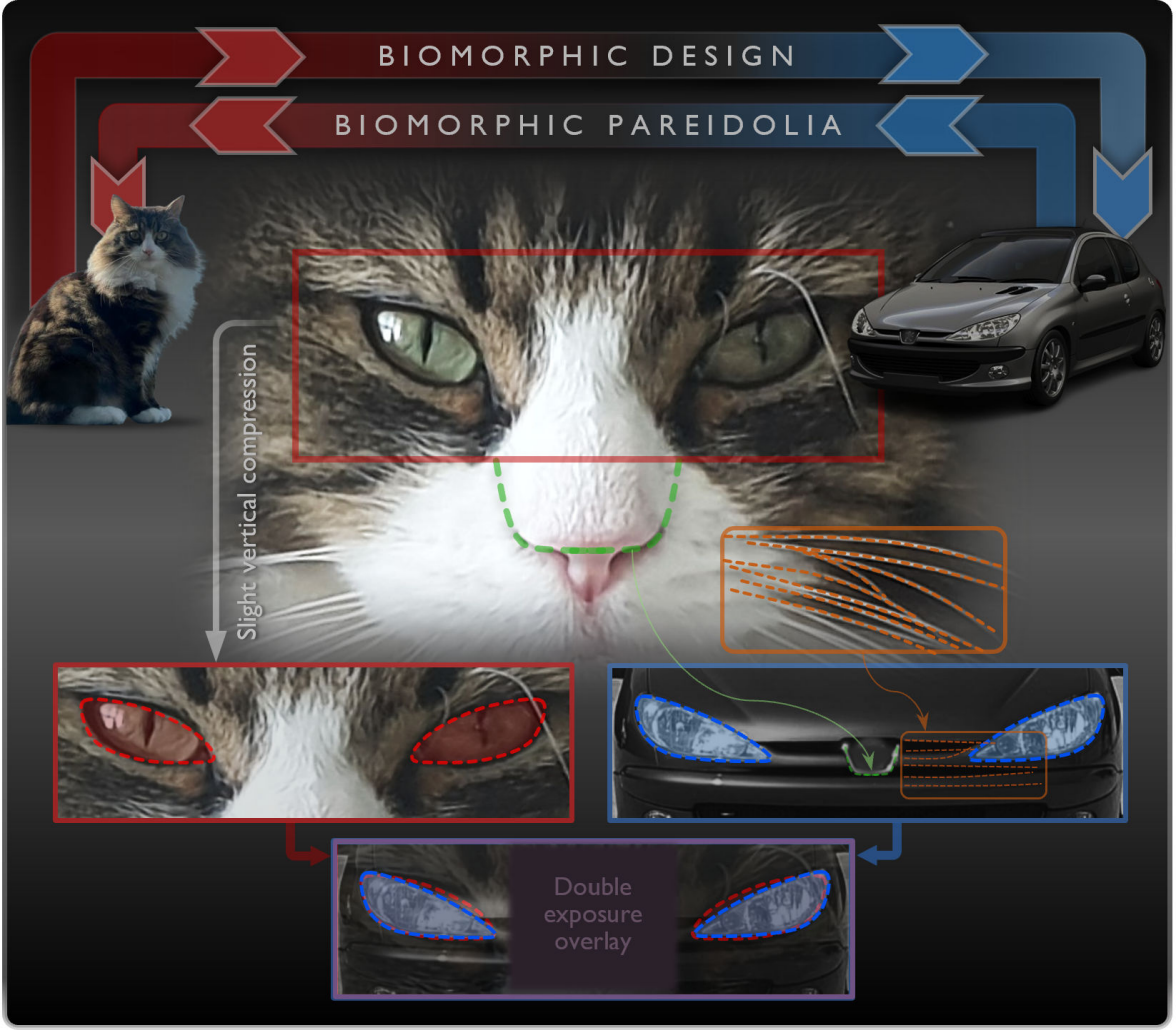
Biologically inspired features in the exterior design of the Peugeot 206, illustrating ‘biomorphic design’ versus ‘biomorphic pareidolia’.

A notable example is the Peugeot 206 (1998−2009), whose front-end design bears a distinct resemblance to the facial features of a typical cat. Eric Berthet, the 206’s exterior designer, stated: ‘*I wanted to create something that was feline and very fluid, but at the same time friendly*’ [80,81]. As shown in figure 11, by slightly compressing the vertical dimensions of a cat’s face and overlaying a semi-transparent image of the cat’s eyes onto a semi-transparent front-end image of the vehicle, we reveal a remarkable similarity between the facial expressions of the two images. Importantly, regardless of the extent of biological inspiration drawn from feline shapes, pareidolia may lead one to perceive feline-like forms or features in the design.

## 3. A fundamental limitation of bioinspiration in automotive design

Nature-inspired design has been a mainstream paradigm for innovations and inventions for centuries. While it is difficult, if not impossible, to find biological equivalents for most man-made machines, some do have ‘natural analogues’ [82]. It is believed that the creation process of such machines is inevitably accompanied by ‘some reference’ to their corresponding natural analogues. For certain ideas, the natural analogue of a conceived invention is readily available in nature, leading an inventor to the ambition of ‘copying’ that natural analogue. A well-known example of such an analogy is the idea of human flight compared with the flight of birds, which inspired Lilienthal to write the famous and influential book *Birdflight as the Basis of Aviation* [82,83]. However, it took several decades for the engineers of flying machines to conclude that directly copying bird flight was not the correct approach to designing aeroplanes.

Over millions of years of evolution, creatures have developed their modes of mobility for survival, characterized by their terrain and mode of locomotion. Most ground vehicles have been designed based on the wheel-based design paradigm, although such a paradigm is rarely found in the anatomy of living organisms. Apart from those organisms that have rotary internal parts, there are very few known creatures that use rotating motion as a mode of locomotion [84].

The rarity of wheeled organisms has been a point of argument. It is widely believed that the primary reason for the absence of mechanisms for wheeled locomotion in creatures is the difficulty of transmitting nutrients across rotary joints, an intrinsic limitation in the developmental evolution of organisms with such a form of locomotion. However, an even more critical issue lies in the mechanics of locomotion itself. Although wheeled movement is energy-efficient on ‘suitable’ terrains, it becomes ineffective on most natural terrains with abrupt changes in local curvature and obstacles. As a general rule, a wheel rolling on a flat surface cannot navigate a step change in elevation that exceeds its radius [84,85]. Notably, it is estimated that more than half of the Earth’s surface is inaccessible to traditional vehicles with wheels and tracks [86,87].

On the other hand, designing dynamically stable and efficient tetrapod-like vehicles presents considerable engineering challenges, making wheeled counterparts much simpler and more cost-effective by comparison. Furthermore, beyond mechanical efficiency—as a mode of transportation on suitable roads or tracks—the wheel-based paradigm offers continuous, smooth motion, ensuring safety, comfort and enjoyment for drivers and passengers. Consequently, the legged locomotion paradigm has remained an impractical option for human transportation. Notably, while wheeled locomotion is not a paradigm found in nature, it has become the dominant approach in automotive design. As a result, much of bioinspiration in automotive design has focused on leveraging the aerodynamic efficiency of biological forms, with wheels integrated into the vehicle’s body, as described in §2.1.

**Figure 12.**
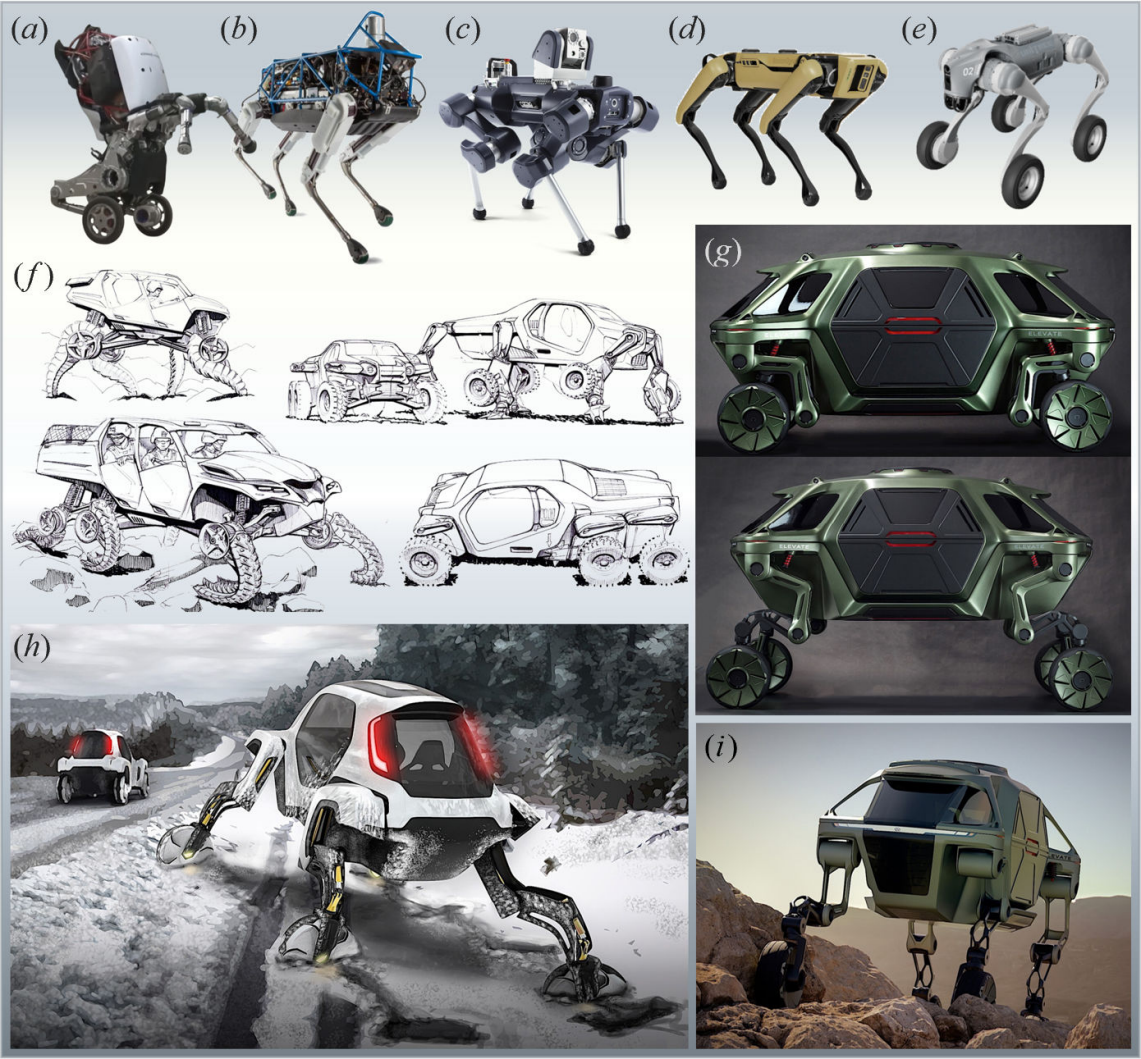
Quadruped robots capable of navigating challenging terrain. (*a*) Boston Dynamics’ Handle [90]. (*b*) Boston Dynamics’ Spot Classic [90]. (*c*) ANYbotics’ impact-protected robot ANYmal X [91]. (*d*) Boston Dynamics’ Spot [92]. (*e*) Unitree’s Go2-W wheeled robot dog. (*f–i*) Hyundai Elevate walking car, from concept sketches to conceived applications in challenging terrain (adapted from [93–96]; courtesy of Hyundai CRADLE SF).

It is worth noting that recent advances in AI and automated control systems have led to the development of successful legged robots, such as quadruped robots [88,89], examples of which are shown in figure 12*a*–*e* (for more examples, see [97–101]). Similarly, mimicking the locomotion of various animals, including different species of fish and birds, has only recently become feasible due to advancements in fields such as materials science and soft robotics (e.g. [102–104]).

In 2019, in an effort to push the boundaries of conventional vehicles, Hyundai introduced the Elevate concept—an electric car with grasshopper-inspired, multi-joint robotic legs. The concept was developed by Hyundai in collaboration with its Centre for Robotic-Augmented Design in Living Experiences (CRADLE) and the industrial design consultancy Sundberg-Ferar. Popularly known as the ‘walking car’, the vehicle was capable of climbing steps, navigating challenging uneven terrains and jumping over gaps [93–96] (figure 12*f*–*i*). However, such innovations have had very limited applications in the automotive industry and, therefore, have not been a major focus of research and development.

Focusing on conventional four-wheeled ground vehicles for the remainder of this study, the next section explores the relationship between form and function in automotive exterior design.

## 4. Formal and functional streamlining in design

### 4.1. Style as a source of form

The prominent industrial designer W. D. Teague (1883−1960), a major figure in the development of the streamlining movement [105], formulated a general theory of industrial design. He proposed that there is a ‘right form’ for an object, historically evolved through a process of trial and error. According to his theory [106], there are six sources of form (SoF), which we denote as SoF-1 to SoF-6 in this study. Here, we briefly outline the first three sources of form (a complete diagram of Teague’s framework is available in [18]).

The first source of form, SoF-1, refers to *fitness*, which asserts that the final form of any man-made object must evolve naturally to fit its intended function, constituting materials and manufacturing techniques. The second source of form, SoF-2, addresses the *laws of relationships*, which pertain to the composition of elements such as lines, areas, shapes, colours and masses. Importantly, these relationships arise from the interactions of these elements rather than being the inherent properties of the individual elements. The central role of these laws is to achieve a state of ‘unity’ by establishing a ‘rhythmic structure’ through the use of appropriate ’proportions’.

In Teague’s framework, the third source of form, SoF-3, is *style*. He opposed any active attempt to apply a style to a design, or to identify it with specific forms, as he believed style to be a continuously evolving entity until it has ‘ceased to live and change’. Teague argued that an ‘authentic style’ emerges naturally as the result of an appropriate response to a specific design problem rooted in a genuine need at a particular time. Through this process of evolution, the designer’s work would integrate into a ‘satisfactorily unified pattern’, inevitably spreading a ‘degree of harmony’ and ‘family likeness’ across their designs. This coherence arises mainly from the frequent recurrence of certain common characteristics, which distinguish these design works from those of any previous era.

### 4.2. Streamlining as a design principle versus a style

‘Style’, as characterized by Teague as ‘authentic style’ in his SoF framework (described in §4.1), is exemplified in the streamlining approach to design during the first half of the twentieth century. Rooted in aeronautics, streamlining was developed and popularized by industrial designers as a style that was both functional (i.e. aerodynamically efficient) and organic (i.e. inspired by natural forms like bird and fish bodies).

Notably, streamlining gradually influenced the design of non-vehicle artefacts, structures and products, eventually evolving into a dominant style known as *Streamline Moderne*. Emerging in the 1930s as ‘a revival of Art Deco’ [107], this style was considered to be ‘symbolic of the dynamic twentieth century, of speed and machines, fast motor cars, railway trains, and steamships’ [108]. I. F. Clarke [109] describes Streamline Moderne as ‘the American version of the machine aesthetic that prized the fluid lines, smooth surfaces and rounded contours of the industrial designers and car manufacturers’.

We classify streamlining into three categories based on the type of artefact:

(i) as a *bioinspired, functionality-driven design principle*, utilized in the design of aircraft (and subsequently trains) for aerodynamic drag reduction;
(ii) as a *functional-aesthetic style*, applied to the design of ground vehicles for aerodynamic drag reduction as well as for stylistic purposes;
(iii) as an *aesthetic style*, applied to the design of non-vehicles, primarily for stylistic purposes.

**Figure 13.**
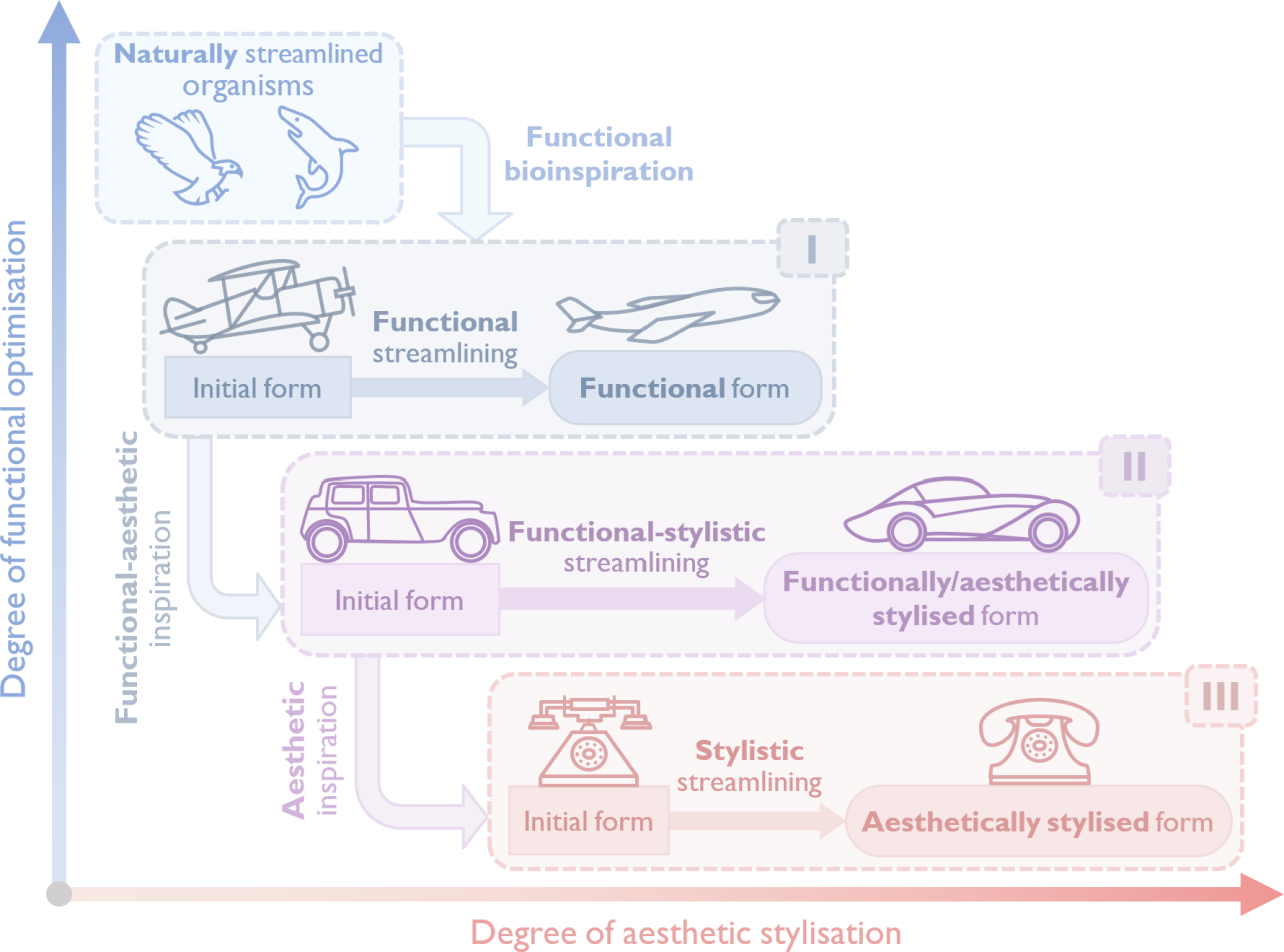
Classification of the streamlining philosophy.

The proposed classification of the streamlining philosophy is illustrated in figure 13.

The next section will focus particularly on the evolution of design methodologies in automobile exterior design.

## 5. Evolution of automobile design methodologies

Automobiles are complex engineering systems whose design and development involve many different criteria. However, their aerodynamic development process, which significantly influences vehicle exterior design, is typically a closed-loop iteration involving three key considerations: aesthetic attributes, packaging requirements and aerodynamic performance [110].

The traditional process of automobile exterior design is a collaborative effort between exterior designers and aerodynamicists, balancing aesthetics, functionality and performance. In this section, we first examine the legacy and current paradigms of this process, exploring how design methodologies have evolved over time. We then envision the future of automobile design and development in the AI era, highlighting emerging technologies, data-driven approaches and the potential for AI-driven generative design to reshape the industry.

### 5.1. Legacy and current paradigms

Since the early twentieth century, streamlining has significantly influenced the design methodology of ground vehicles. Initially, aerodynamic design for vehicle bodies was largely driven by the desire to achieve higher top speeds. Later, until the 1970s, exterior design was primarily a ‘*statement of style and fashion with little regard for the economic benefits’* [111]. In other words, in the automobile industry, streamlining was more often a ‘*matter of styling than a real desire to reduce drag’* [112]. Market resistance to the unconventional appearance of genuinely aerodynamic designs, which would have reduced drag, contributed to this trend.

The economic crisis of the early 1970s, along with rising fuel prices, sparked a demand for more economical vehicles with significantly higher fuel efficiency. Since then, aerodynamic performance, engine efficiency and lightweight construction have emerged as the three primary goals of automotive engineering [111]. Aerodynamic performance itself is influenced by three key factors: (i) airflow around the body, (ii) airflow through the body, and (iii) the airflow field within under-bonnet components [113]. Of these, the first factor has been the most relevant to the body shape design and styling of automobiles.

**Figure 14.**
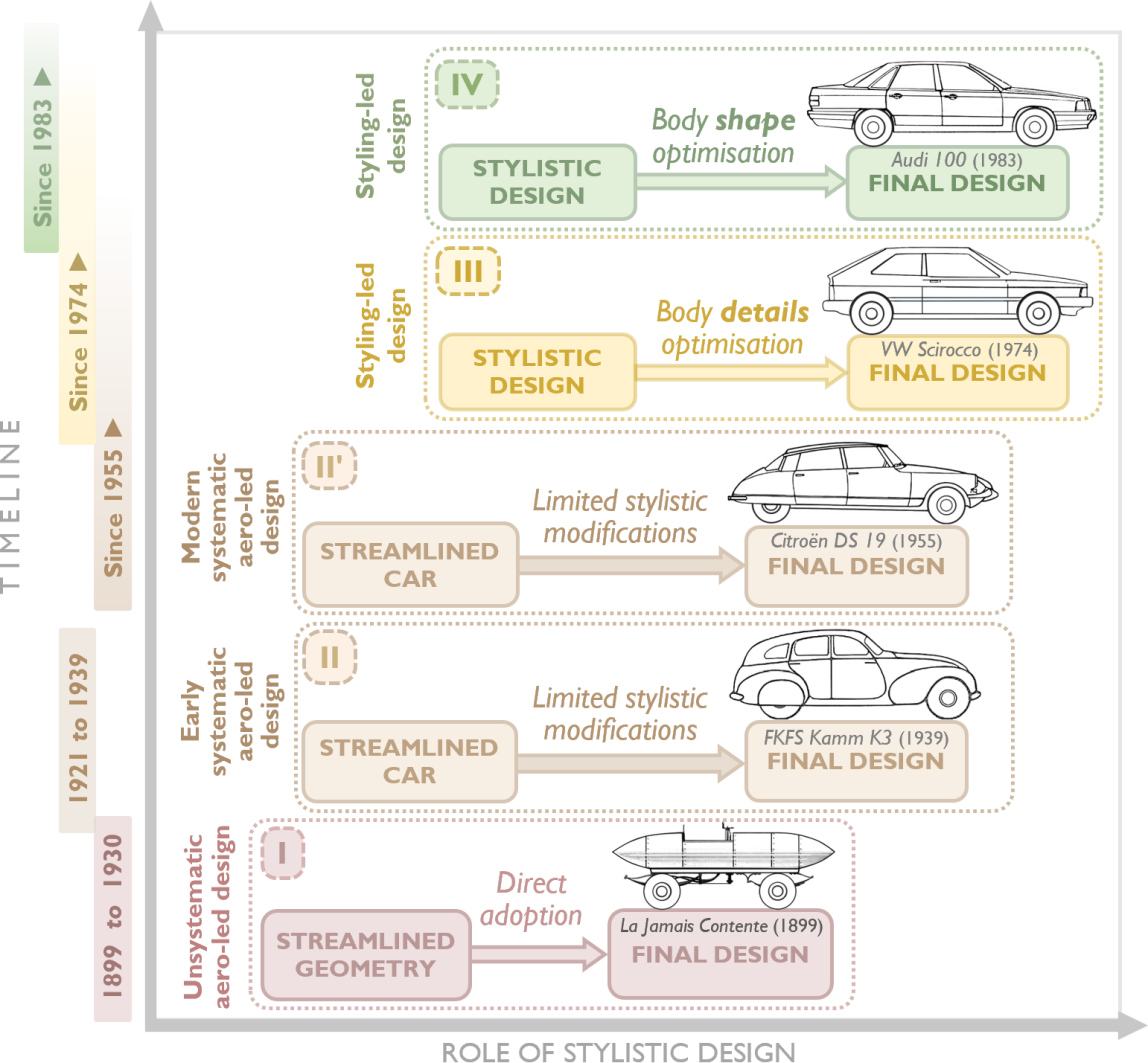
Influence of the streamlining philosophy on the design methodology of passenger cars.

According to Hucho *et al*. [114], the history of automobile aerodynamics can be divided into four ‘chronologically indistinct’ phases, which we examine here from a design perspective, as illustrated in figure 14.

In the first phase, spanning from the late 1890s to around 1930, car builders attempted to directly adopt well-known streamlined geometries, previously developed by aeronautical and marine engineers, in the design of passenger ground vehicles. These efforts were largely unmethodical and primarily aimed at achieving record speeds on land. However, due to the poor road conditions and limited power of internal combustion engines at the time—factors that imposed additional operational constraints—these attempts did not result in practical solutions. We refer to this approach, used in the first phase, as the *unsystematic aerodynamics-led design method*.

In the second phase, drawing on recent advances in aeronautics, aerodynamicists sought to develop the so-called ‘streamlined car’ using systematic engineering approaches. However, because the overall vehicle form was dominated by drag minimization, this led to excessively long and slender body shapes that were incompatible with practical needs, such as adequate internal space for passengers. Nonetheless, an aerodynamic breakthrough emerged during this period, known as the ‘Kamm-tail’, ‘Kamm-back’ or ‘K-back.’ The ‘blunt rear-end shape’ of K-back bodies allowed airflow to remain attached to the body surface for a relatively long time before detaching abruptly at the rear end with a reduced cross-sectional area. This innovation made it possible to achieve a streamlined car without excessive length, providing improved headroom for backseat passengers while maintaining sufficiently low aerodynamic drag [113,114]. The use of Kamm-tails remains an effective and practical design approach for enhancing aerodynamic efficiency in the automotive industry.

We refer to this approach as the *systematic aerodynamics-led design method*. From a form–function relationship perspective, the design process associated with this method was dominated by creating a form that prioritized a single primary function—minimizing aerodynamic drag—by achieving the theoretically ideal shape of a ‘streamlined car’. Subsequently, passengers and other vehicle components were accommodated within this predefined geometry, necessitating significant compromises and sacrifices. This approach can be summarized as follows: *form* first follows the *primary function f*, then is adjusted to address *secondary functions g*_1_, *g*_2_, … , *g_n_*, where *f* represents aerodynamic drag minimization, and *g_i_* (*i* = 1,2,..,*n*) denotes a set of *n* secondary functions, including adjustments for efficient packaging, passenger safety and passenger comfort.

**Figure 15.**
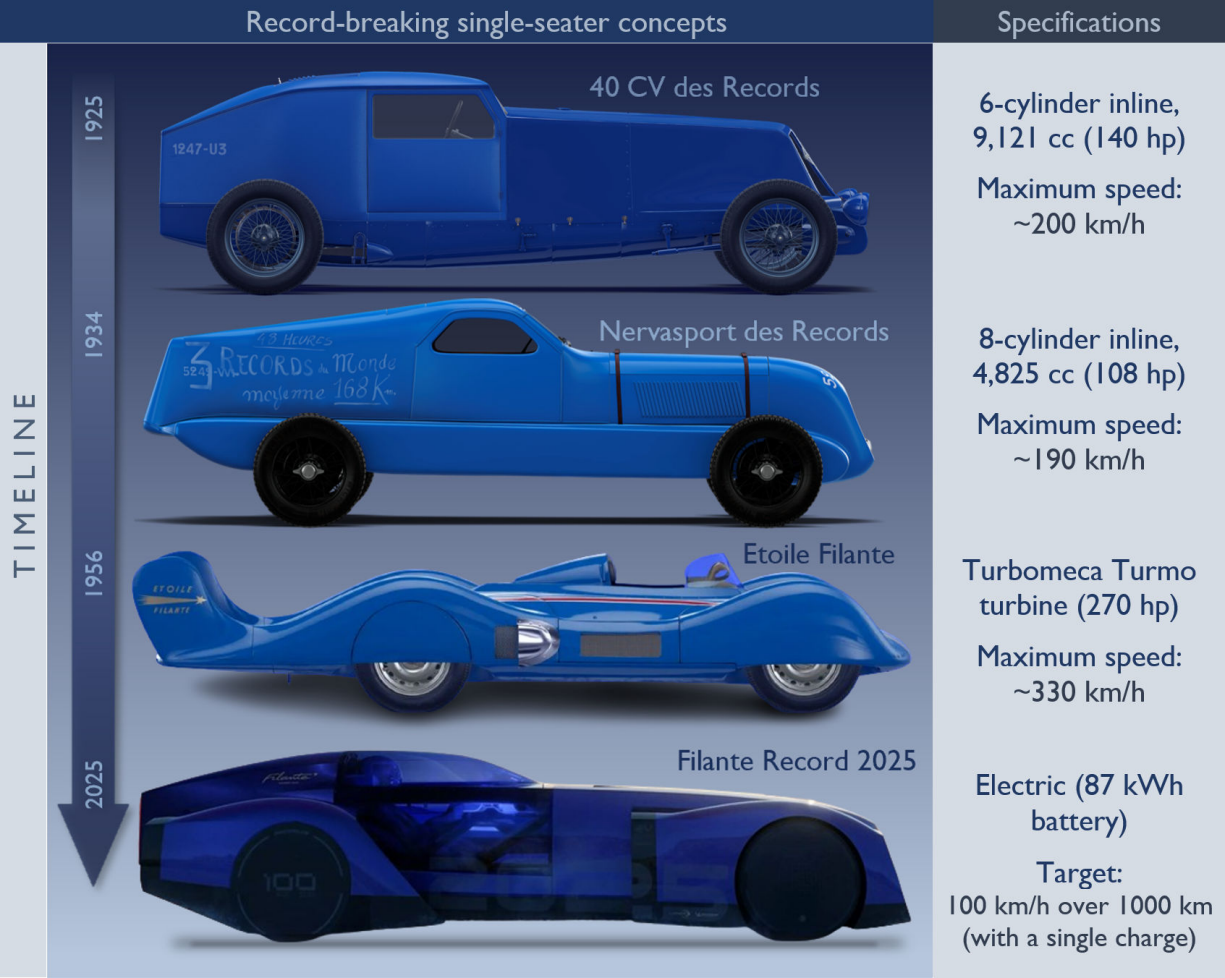
Renault’s historical record-breaking cars and their specifications (adapted from [115–117]).

It is important to note that minimizing aerodynamic drag may still be the primary function of exterior design in certain concept cars. For example, Renault recently introduced the electric demo car, Filante Record 2025, to push the limits of energy efficiency and set new records for fuel consumption and range. The car was inspired by the speed record-breaking 40 hp single-seater from 1925, the Renault 40 CV des Records. Other historical attempts by the manufacturer include the Renault Nervasport des Records (1934) and the highly streamlined Renault Étoile Filante (1956). Figure 15 presents the profile views of these vehicles along with their respective technical specifications [115–117].

The third phase employs a method developed in the 1960s [118], which was first introduced as a systematic approach in a 1976 article by Volkswagen [114]. Unlike the two preceding methods, this approach does not directly deal with streamlined forms. Instead, a chosen ‘*stylistic design*’, created by vehicle designers, serves as the starting point for aerodynamic development, where geometric modifications to the shape must be made ‘*within the styling concept*’ [113].

Using this method, based on a physical or virtual three-dimensional model of the proposed stylistic design, various components are optimized through systematic modifications, followed by aerodynamic analyses of the altered forms. This process involves adjusting details such as surface curvature, taper and spoilers to prevent flow separation or to control separation to achieve drag minimization. The result is an aerodynamically optimized version of the design originally created by the vehicle designers [113,114].

This method is based on the postulate that ‘*the styling concept of a vehicle must be accepted as it stands*’ [114]. It presented a significant challenge for aerodynamicists, encapsulated in the following directive: ‘*Make it aerodynamic but don’t change the style*’ [118]. We refer to this approach as the *styling-led design method*. Examining this modern process for the exterior design of passenger cars, we propose the following expression to describe the relationship between form and function: ‘form first follows style, then is refined by function’. This concept can be generalized to design problems involving multiple functions, where each function requires meeting specific functional demands, as follows: ‘*form* first follows *style*, then is refined by *functions*’.

In the late 1970s, General Motors (GM) developed a series of compact economy cars based on its ‘X platform’, which were marketed from the 1980 to the 1985 model years [119]. For these vehicles, collectively referred to as ‘X-Cars’, GM implemented a systematic aerodynamic programme featuring a novel approach to drag reduction [120] for the front-wheel-drive models of each of its divisions: the Chevrolet Citation, Pontiac Phoenix, Oldsmobile Omega and Buick Skylark.

Unlike Volkswagen’s approach described earlier, where the vehicle’s appearance was established first and then optimized for low drag, GM initiated aerodynamic development early in the design process, before finalizing the vehicle’s basic appearance. Importantly, wind tunnel test results played a crucial role in shaping and fine-tuning the overall appearance of the X-Cars [120].

**Figure 16.**
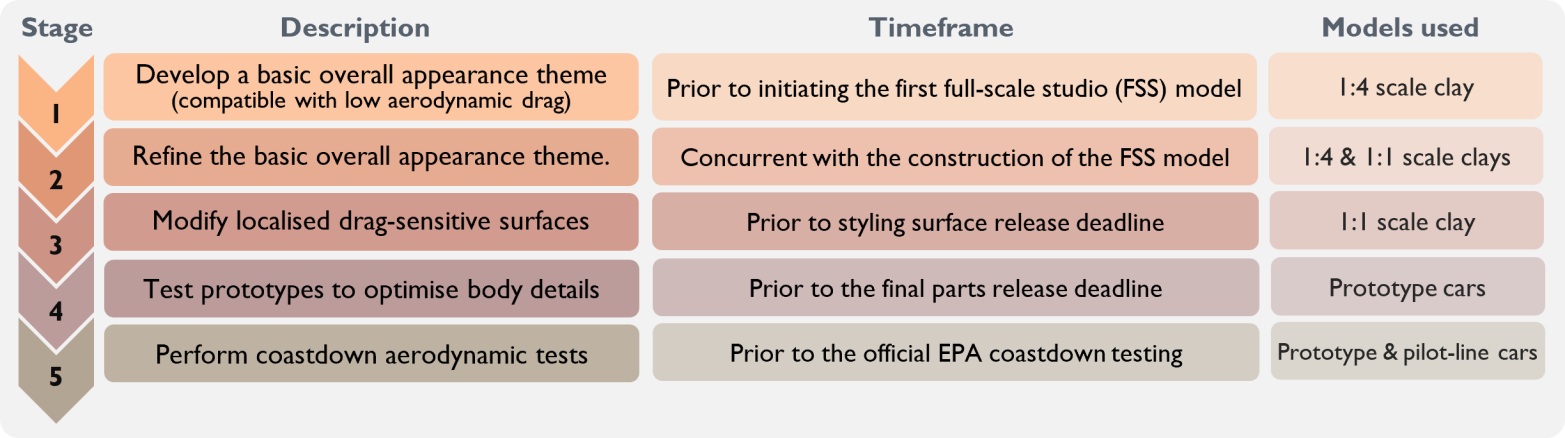
Five stages of the aerodynamics programme for GM’s 1980 X-Car project (adapted from [120]).

As illustrated in figure 16, GM’s 1980 X-Car aerodynamic programme encompassed five stages. It began with the development of a ‘basic overall appearance theme’ compatible with low aerodynamic drag, using 1 : 4 scale clay models. This was followed by gradual refinement of the overall appearance theme, modifications to localized drag-sensitive surfaces, prototype testing to optimize body details, and, finally, road coastdown testing using pilot-line cars [120].

This approach was first applied to GM’s production X-Cars in 1980, achieving a drag coefficient lower than that of any previously tested domestic or imported family car [120]. However, despite initial market success, the X-Car project ultimately resulted in failure and became associated with one of the largest recalls in the industry due to various engineering flaws, most notably defective braking systems [121,122].

Parallel to GM’s work on applying their method to the design of X-Cars, VW was developing a similar method, formalized by Hucho [123] as the fourth phase of automotive aerodynamic development. The strategy defining this phase is to start with a streamlined ‘basic body’ created by aerodynamicists. This basic body already incorporates the key dimensions (overall length, height and width) of the finished vehicle. During this stage, scale models are often employed to reduce expenses and simplify model handling.

Using the basic body, a ‘basic shape’ is first created by systematically varying geometric parameters. This shape incorporates the primary features of a passenger car body, such as a rounded front and a curved windshield. Typically, the drag coefficient of this basic shape is only slightly higher than that of the basic body. The next step is to develop a ‘basic model’ from the basic shape by incorporating all the technical characteristics of a practical car. The airflow around the body becomes less smooth due to additional details such as window recesses, joints, beads, suspension parts, the exhaust pipe and the muffler. Furthermore, the airflow through components like the radiator and wheel wells results in an additional increase in drag. Once this basic model is complete, it is handed over to the design department to develop a ‘styling model’. During the styling process, any increase in drag depends heavily on the skill of the designers and the level of cooperation between them and the aerodynamicists [123,124].

**Figure 17.**
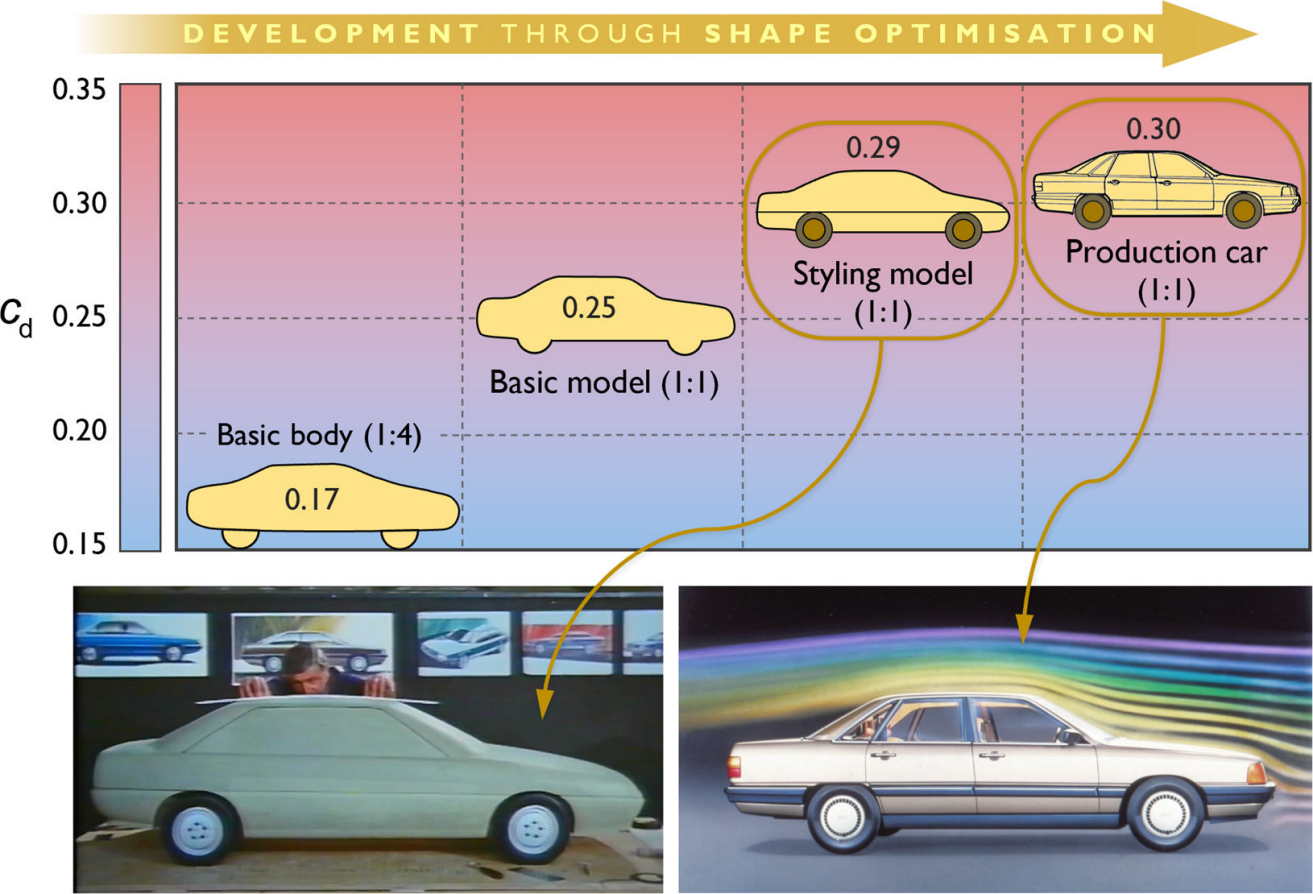
Development of the third-generation Audi 100 (launched in 1982) through the shape optimization process (adapted from [123,125,126]).

The development of the third-generation Audi 100 is a notable example of design refinement using the aforementioned shape optimization strategy [123]. Launched in 1982, the Audi 100 (figure 17) achieved a drag coefficient of 0.30, making it the most aerodynamically efficient production saloon in the world at the time. For context, as of 2024, the A6 Sportback e-tron holds the title of Audi’s most aerodynamically efficient vehicle, with a drag coefficient of 0.21 [125,127].

**Figure 18.**
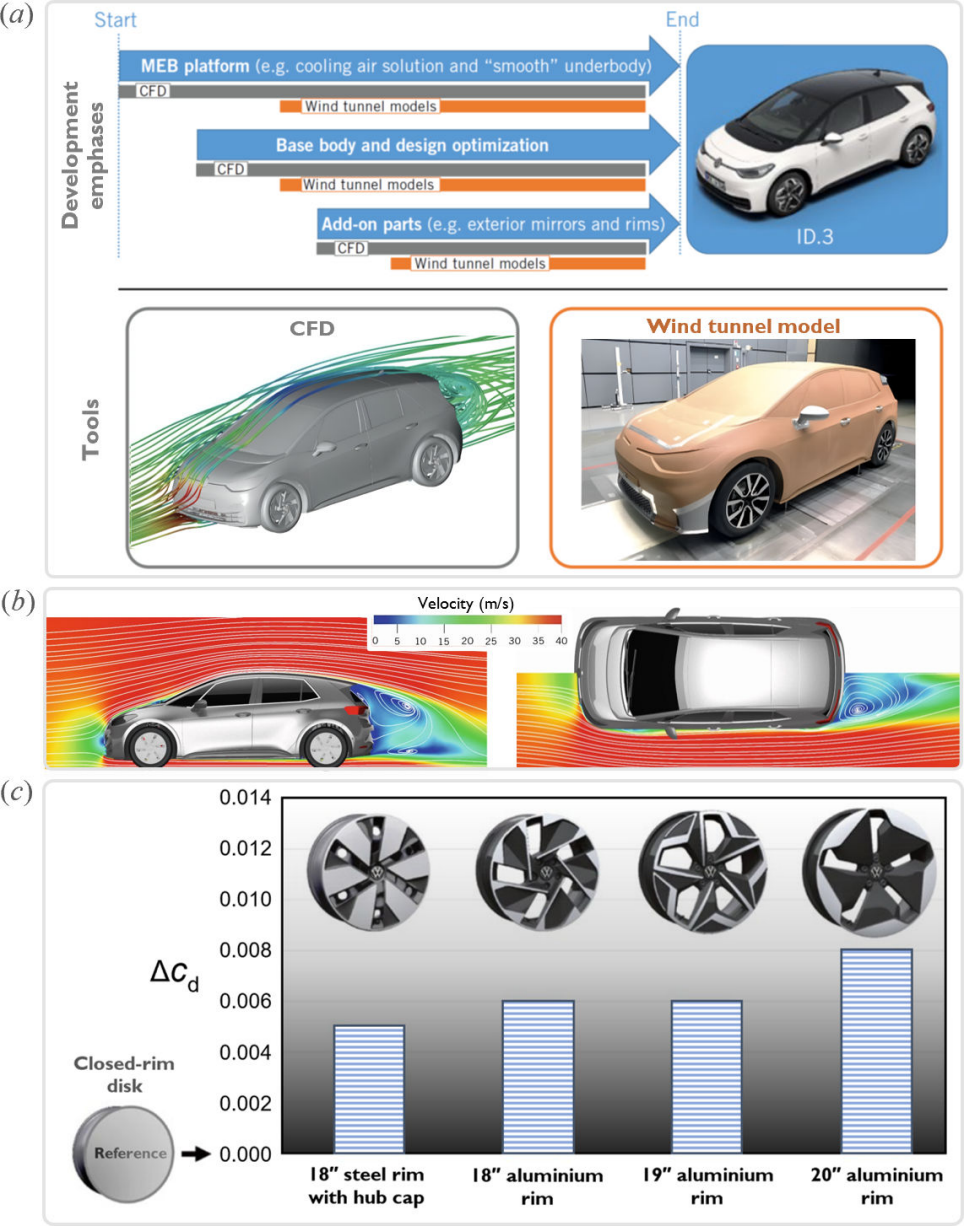
Aerodynamics of the Volkswagen ID.3 electric car. (*a*,*b*) Aerodynamic development phases and tools (© Volkswagen). (*c*) Differences in drag coefficients for various design candidates of the ID.3 rim, measured relative to a fully closed disc rim (© Volkswagen) (adapted from [128]).

This method remains widely used as the primary design and development approach in the automotive industry, employing two key analysis and experimentation tools: computational fluid dynamics (CFD) simulation and wind tunnel testing. For example, figure 18 illustrates the phases and engineering tools involved in the aerodynamic development of the Volkswagen ID.3 electric car. Using a highly detailed CFD model and a systematic approach in the early stages of development, Volkswagen aerodynamicists optimized not only the ‘basic styling shape’ but also add-on components such as side mirrors and rims. These numerical investigations were complemented and validated through detailed, modifiable wind tunnel models. This aerodynamic design process enabled the ID.3 development team to achieve a remarkable drag coefficient of 0.26, substantially increasing the range of this electric vehicle [128].

**Figure 19.**
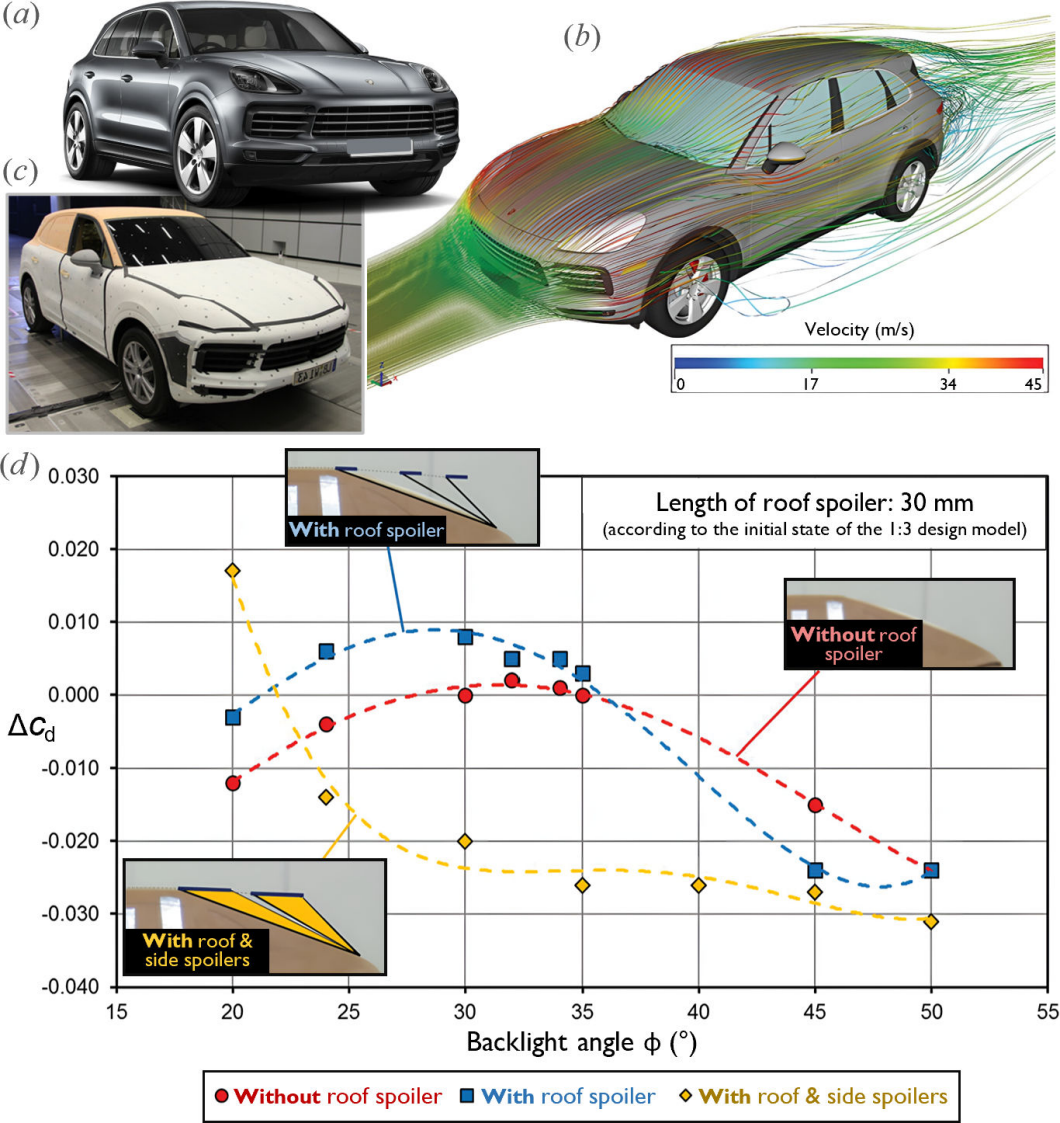
Aerodynamic development of the third-generation Porsche Cayenne. (*a*) Production model. (*b*) CFD simulation of external and internal flow. (*c*) Full-scale wind tunnel model. (*d*) Effect of the backlight angle and the roof spoiler configuration on aerodynamic drag coefficient variation (adapted from [129]).

The aerodynamic development of the third-generation Porsche Cayenne (figure 19), developed between 2012 and 2017, involved creating several full-scale models and up to four one-to-three scale styling models, produced by the design department for aerodynamic evaluation. These models for the new Cayenne were tested both experimentally in Porsche’s one-to-one and one-to-three wind tunnels and through flow simulations, with a one-to-three model of the previous generation serving as a benchmark [129].

**Figure 20.**
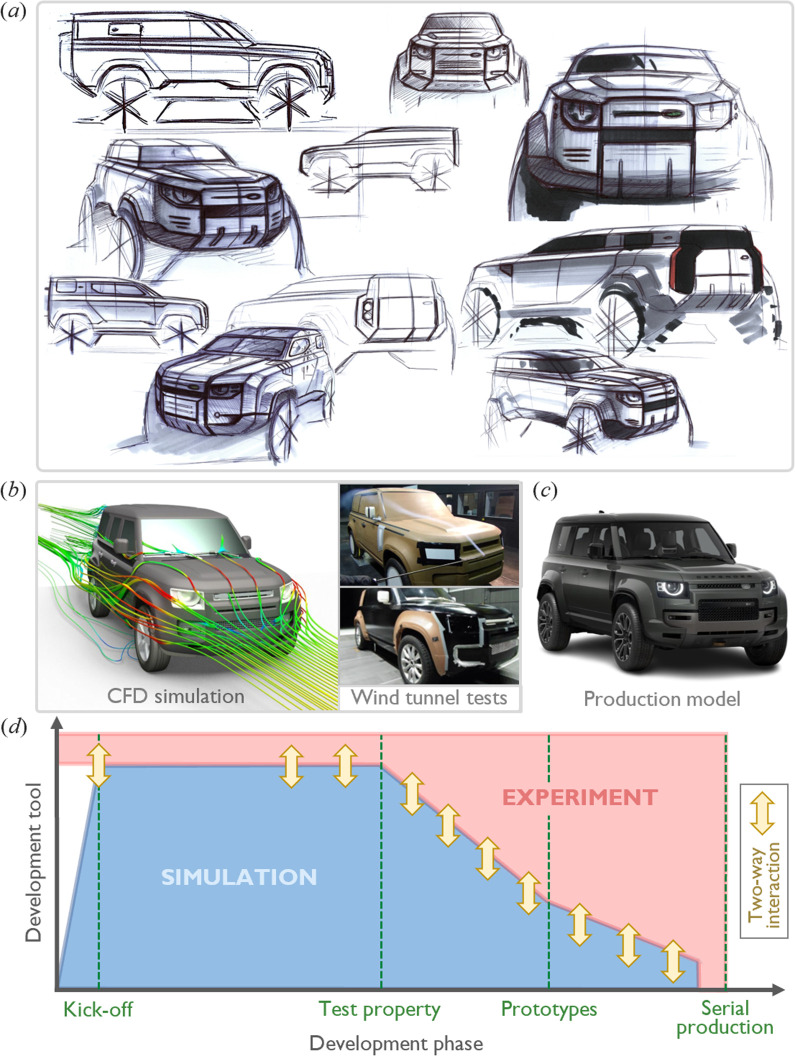
Design and development of the new Land Rover Defender 110. (*a*) Presentation of official design sketches (adapted from [130]). (*b*) Overview of the aerodynamic development process, including CFD simulations and wind tunnel tests (adapted from [131]). (*c*) Production model. (*d*) Diagram of the iterative aerodynamic development process, characterized by a continuous interplay between simulations and physical experiments (adapted from [131]).

A more recent example is the development process of the new Land Rover Defender 110, as illustrated in figure 20. As a historical symbol of strength and toughness, designing a Defender fit for the twenty-first century posed the challenge of preserving key elements of its design vision and historical identity. From an aerodynamic perspective [131], the primary constraints and challenges included:

(i) *Vertical surfaces*: the steep angles of the front and rear surfaces, including the windscreen, increased drag, causing aerodynamic inefficiencies.
(ii) *Squared A-pillars*: the sharp, squared-off sections of the A-pillars disrupted smooth airflow along the sides of the vehicle.
(iii) *Flat bonnet and roofline*: a lack of curvature in the bonnet and roofline further disrupted airflow, making it challenging to achieve optimal aerodynamic performance. More specifically^1^:
  - A flat bonnet makes it more difficult to avoid flow separation during the transition from the more vertical front face and may lead to earlier flow separation as the windscreen is approached, along with a stronger lateral vortex system at the base of the windscreen.
  - For the roof, the main issue is the missed opportunity to promote additional pressure recovery by sloping it downward towards the rear. The goal is to maximize pressure at the roof trailing edge separation which, in turn, increases pressure at the base, reducing drag. (This is the same mechanism exploited by roof trailing edge spoilers.)
(iv) *No roof spoiler*: the absence of a trailing edge roof spoiler limited the ability to reduce drag or manage airflow at the rear.
(v) *Ground clearance and exposed wheels*: the large ground clearance, combined with exposed wheels and tyres, introduced additional turbulence underneath the vehicle.
(vi) *Robust underbody design*: designed for off-road use, the vehicle required a durable underbody, which constrained efforts to streamline or smooth this area for improved aerodynamics.

Despite these significant constraints and challenges, the integration of numerical simulations and physical tests enabled the team to meet demanding aerodynamic targets by leveraging their expertise and selecting the most effective tools for each project phase. Notably, the aerodynamic development programme for the new Land Rover Defender 110, as illustrated in figure 20, achieved a 48% reduction in drag compared with its predecessor, which had been developed without numerical tools or moving ground wind tunnels. This demonstrates that even bluff, utilitarian road vehicles can achieve significantly improved aerodynamic designs through the effective combination of advanced experimental and computational tools [131]. It is also evident from this figure that CFD was the primary tool in the early concept phases, with a transition to the wind tunnel as the lead tool later in development.

The exterior design process of the Tesla Model S exemplifies systematic design optimization through aerodynamic development [132,133]. Following the overwhelmingly positive response to the Model S concept car, preserving its distinctive styling became a priority while enhancing its aerodynamic performance. Although this limited the scope of aerodynamics-driven design changes, close collaboration between designers and aerodynamicists—facilitated by the integration of the Design and Engineering teams at Tesla Motors—proved to be an effective strategy. Notably, from the earliest design sketches, an aerodynamicist worked closely with the designers, providing support and guidance.

**Figure 21.**
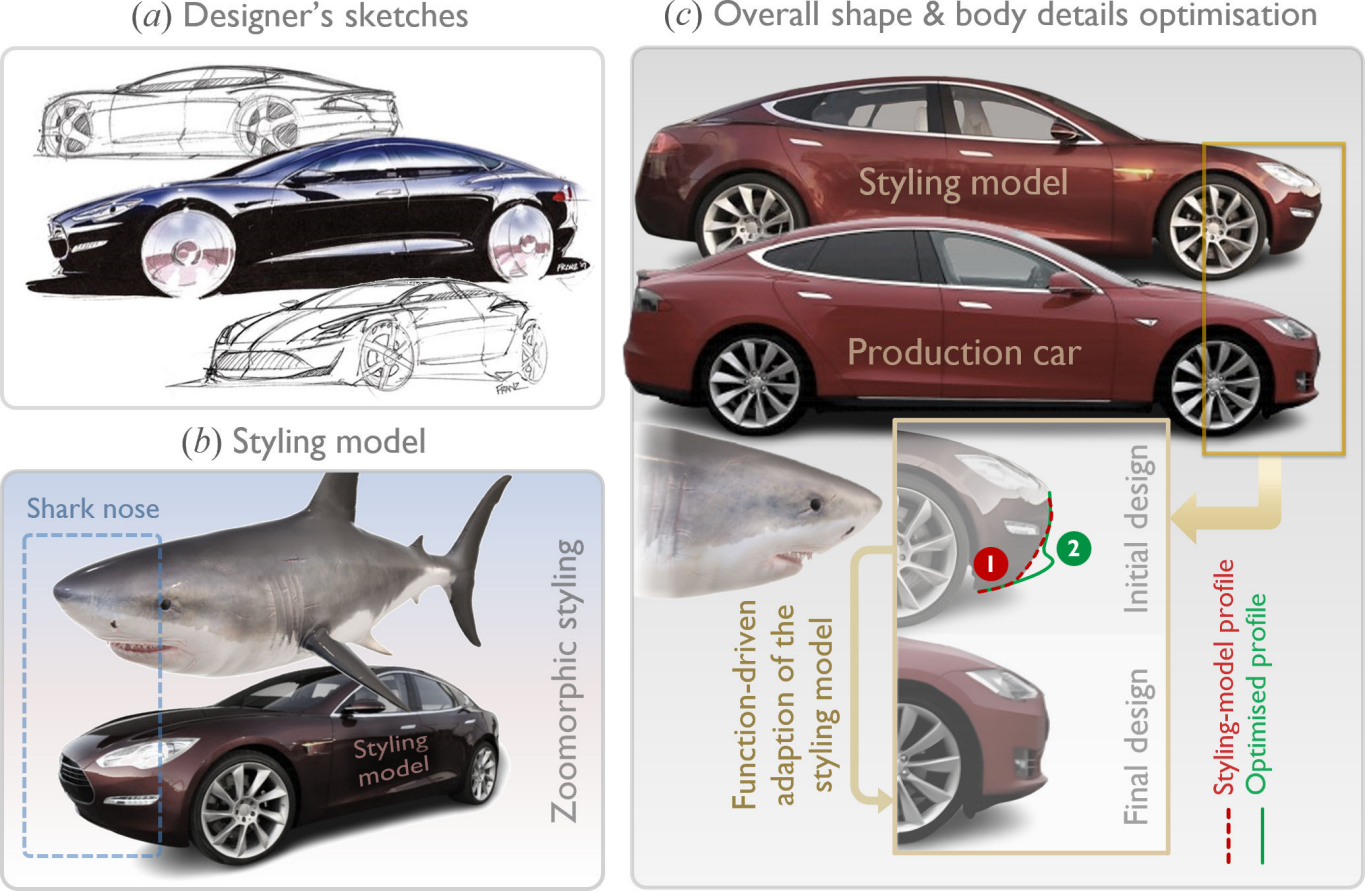
Exterior design process of the Tesla Model S (2012). (*a*) Initial sketches by Franz von Holzhausen (adapted from [134]). (*b*) Resemblance of the styling model’s frontal design to a shark’s nose. (*c*) Optimization of the overall shape and body details, with particular focus on modifying the shark-nose profile of the styling model (photos of the styling model adapted from [132,135]).

Beginning with a styling model based on Franz von Holzhausen’s sketches (figure 21*a*), the aerodynamic development of the Model S followed the traditional approach of optimizing the overall shape before refining the details. This multidisciplinary cooperation, combined with extensive use of CFD, resulted in a 0.07 reduction in drag coefficient between the initial approved styling model and the first wind tunnel model. One significant modification involved softening the so-called ‘shark-nose’ profile [132] at the front by extending the areas beneath the headlights, as shown in figure 21*b*,*c*, exemplifying a *function-driven adaptation* of a biomorphic design. Additionally, at the rear, the vehicle’s perimeter was widened, and the deck lid was elongated.

In recent years, increasingly stringent vehicle emission standards have elevated the importance of aerodynamic design optimization in road vehicle design and development. This shift has led some in the automotive industry to claim that ‘the science of aerodynamics is taking priority over the aesthetics of design’ [136]. To balance the roles of aerodynamics and aesthetics from the early stages of the design process, it is suggested that an integrated styling-aerodynamic studio could be established. In this set-up, designers would receive ‘actionable feedback and insight on how to improve the design’ with regard to the aerodynamic performance of their stylistic concepts, facilitated by highly detailed and accurate computational simulations. This approach has been referred to as ‘simulation-driven design’ [136]. However, we believe that such a term might be misleading, as conventional simulations merely represent aerodynamic properties. This implies a functionalist approach rather than a process in which stylistic design and aerodynamic performance are integrated to achieve a rational balance between the two.

We anticipate that the concept of an integrated styling-aerodynamic studio will be effectively realized within an AI-augmented design environment in the coming years, as outlined in the next section.

### 5.2. Envisioning the future: the fifth phase of automobile design in the AI era

#### 5.2.1. Advances in integrating styling and aerodynamic optimization

Over the past three decades, there have been many attempts to computationally augment the early-stage automotive design process. These efforts include the use of graph theory [137], shape grammars [138] and statistical techniques [139–141], which have been proposed to support design ideation and concept exploration. However, these methods primarily address the formal attributes of designs, leaving the functional aspects to be explored in a separate phase.

Recently, the introduction of machine learning to vehicle design has shown great potential for enhancing the traditional aesthetic design process. For instance, Burnap *et al*. [142] proposed an AI-augmented model to advance aesthetic design. By leveraging deep learning techniques, the model predicts the aesthetic appeal of designs and generates innovative, attractive options. Trained on a large dataset of automotive images, it demonstrates significant improvements in predicting consumer preferences and generating realistic, market-relevant designs. Nevertheless, such initiatives remain focused solely on the formal aspects of vehicle design.

Recent collaborations between Autodesk and Kia Global Design [143], and Autodesk and Hyundai Motor Company [144], highlighted challenges in applying generative AI to automotive design, such as stylistic bias, difficulty in interpreting nuanced text or image meanings, and limited integration with design workflows. To address these challenges, workshops and surveys with automotive designers guided the creation of a diffusion-based AI system. This system enhances concept generation inspired by texts and images, aligning with designers’ practices and interaction preferences to streamline and improve conceptual automotive design processes [144].

Notably, by integrating functional constraints and metrics through physics-based methods, both predictive and generative AI tools are poised to revolutionize the vehicle design process. These tools enable stylists and engineers to collaborate from the earliest stages of a new concept’s creation, facilitating the conception, evaluation and refinement of design iterations with unprecedented efficiency and cost-effectiveness. Moreover, the integration of virtual reality (VR) and augmented reality (AR) is expected to further enhance the effectiveness and efficiency of these processes.

**Figure 22.**
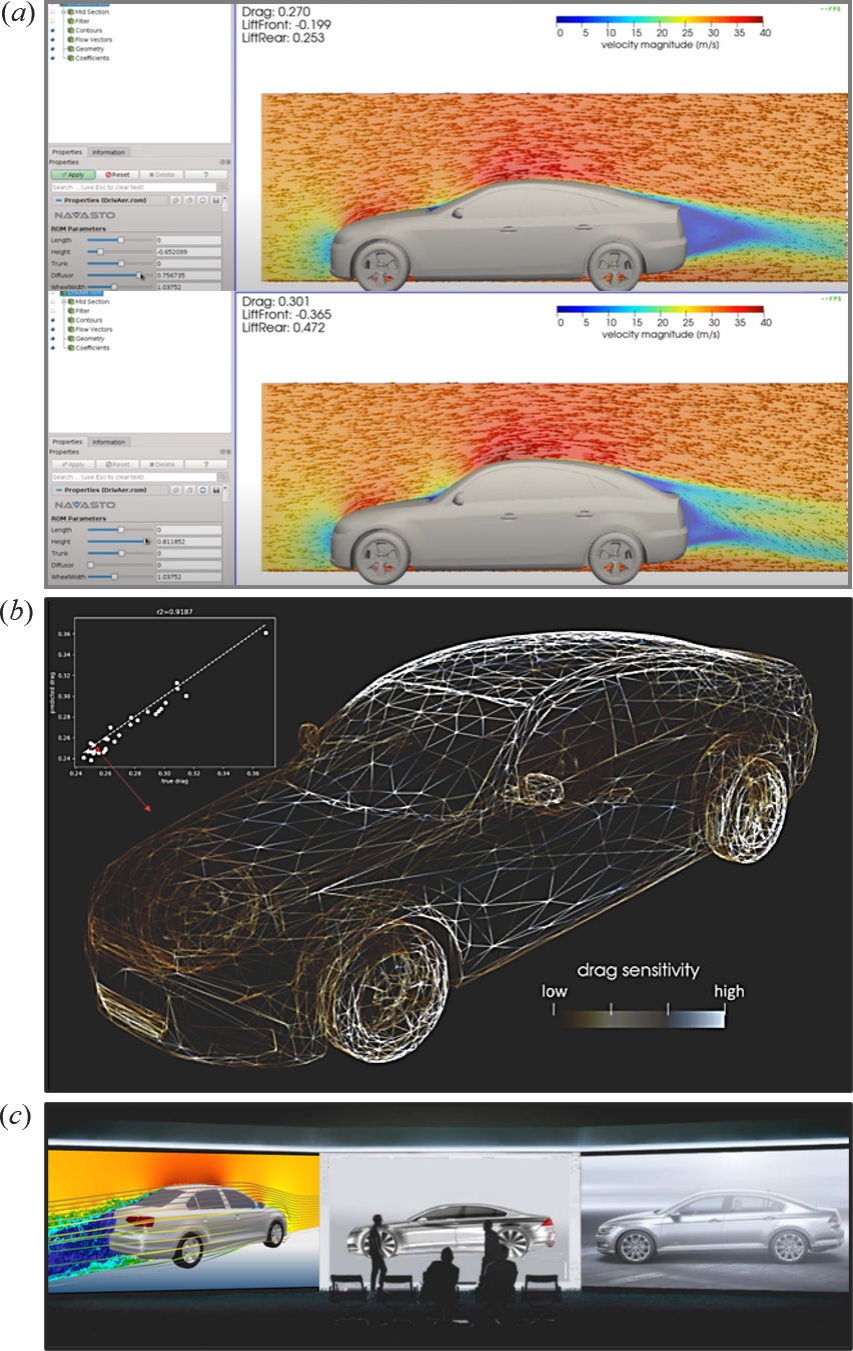
An AI-based aerodynamic simulator. (*a*) Real-time interactive design and aerodynamic optimization. (*b*) Drag sensitivity of a vehicle at various points. (*c*) Illustration of an interactive, AI-augmented collaborative design environment (adapted from NAVASTO [145]).

For example, NAVASTO [145] has recently developed an AI-based aerodynamic simulator. This computational tool enables the prediction of aerodynamic coefficients and the computation of node-wise sensitivities for a given geometric design within seconds, as depicted in figure 22*a*,*b*, respectively. Such a technology has the potential to largely replace many CFD simulations, which are typically computationally expensive and time-consuming. It could also empower the design and development team to make real-time decisions based on immediate feedback in an interactive, AI-augmented collaborative environment, as showcased in figure 22*c*.

**Figure 23.**
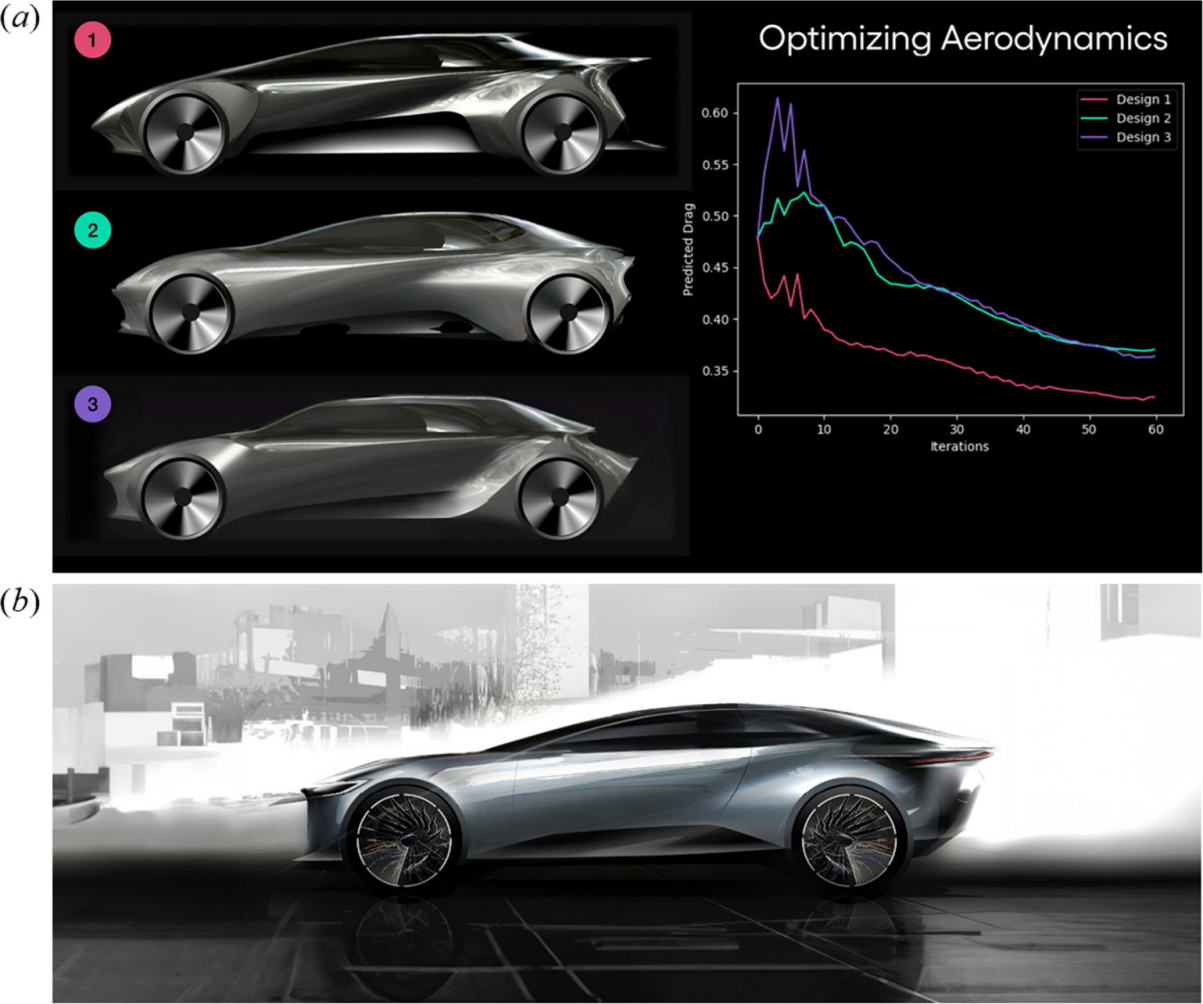
TRI’s new generative AI and optimization technology for vehicle design. (*a*) Aerodynamic drag minimization through successive iterations based on generative AI and parameter inputs from the designer. (*b*) Vehicle design sketch produced using the results of this technology (courtesy of XD: Experimental Design Studio of Toyota, adapted from [147]).

Additionally, early-stage research by the Toyota Research Institute (TRI) has demonstrated that current image generation methods, such as diffusion models [146], can be modified to optimize aerodynamic drag. This allows vehicle designers to generate novel concepts while simultaneously minimizing their predicted drag coefficients, as illustrated in figure 23. This promising outcome suggests that other performance metrics, such as packaging efficiency, could be incorporated in the future [147,148].

Several software companies have developed integrated workflows for styling and aerodynamic simulation, aiming to unify stylistic design with aerodynamic optimization into a single process. For example, PowerFLOW, developed by SIMULIA, is a CFD tool designed to simulate fluid flow around vehicles in real time, providing transient aerodynamic simulations under both idealized uniform flow conditions and realistic wind environments. This enables comprehensive assessment of aerodynamic performance and drag, allowing analysis of various parts of the vehicle [149].

DesignGUIDE, introduced in the 2020 release of PowerFLOW, adds a graphical interface that connects performance metrics to design adjustments, offering stylists intuitive feedback. Using an interactive colour-coded surface map, it provides a three-dimensional representation of the vehicle, showing how specific surface modifications influence aerodynamic performance. For instance, it highlights areas where pulling a surface outward increases drag, while pushing it inward reduces drag. Such insights enable designers and engineers to swiftly evaluate vehicle performance and, when applied early in the design process, guide decisions to balance aesthetics with aerodynamic efficiency [149].

Similarly, Autodesk’s Studio Wind Tunnel Project provides automotive studio designers with advanced simulation insights and guidance by leveraging high-performance computing. This approach streamlines the design process, eliminating iterative feedback loops and enabling significantly faster aerodynamic evaluations at any stage of vehicle development. Integrated directly with Autodesk Alias, it allows designers to access aerodynamic insights without relying on external expertise or resources [150].

#### 5.2.2. Formalization of the fifth phase of automobile design

As explained in §5.1, the history of automobile aerodynamic design has been divided into four chronologically indistinct phases. Here, we propose that a new era is emerging, facilitated by AI, which we identify as the fifth historical phase of automobile design.

**Figure 24.**
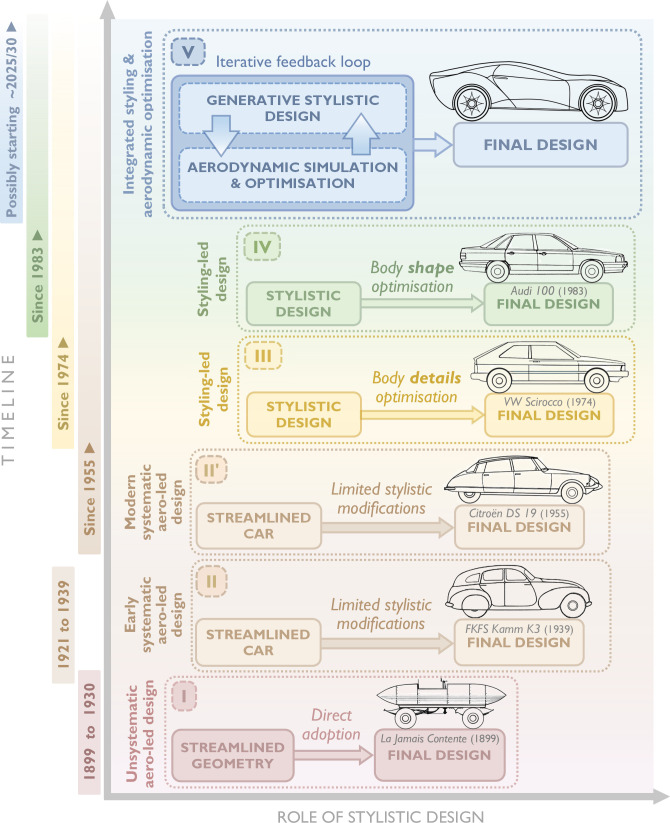
Integrated Styling and Aerodynamic Optimization (InStyAO) proposed as the fifth historical phase of automobile design.

To expand Hugo’s historical classification and formalize this emerging phase, we introduce *Integrated Styling and Aerodynamic Optimization* (*InStyAO*), an AI-augmented method that incorporates concurrent generative stylistic design, aerodynamic simulation, evaluation and optimization. This method enables form and function to be concurrently evaluated, modified and updated through a feedback loop, resulting in a highly optimized final design. This phase is depicted as ‘Phase V’ in the upper part of figure 24.

This approach to design can be summarized as follows: ‘form(s) and function(s) mutually inform and modify each other towards an optimized compromise’. It represents a specific instance of a broader design methodology, *Integrated Styling and Engineering* (*InStylEng*), in which, alongside aesthetic considerations, multiple functional requirements—such as aerodynamic performance and packaging efficiency—are simultaneously addressed in the design process.

**Figure 25.**
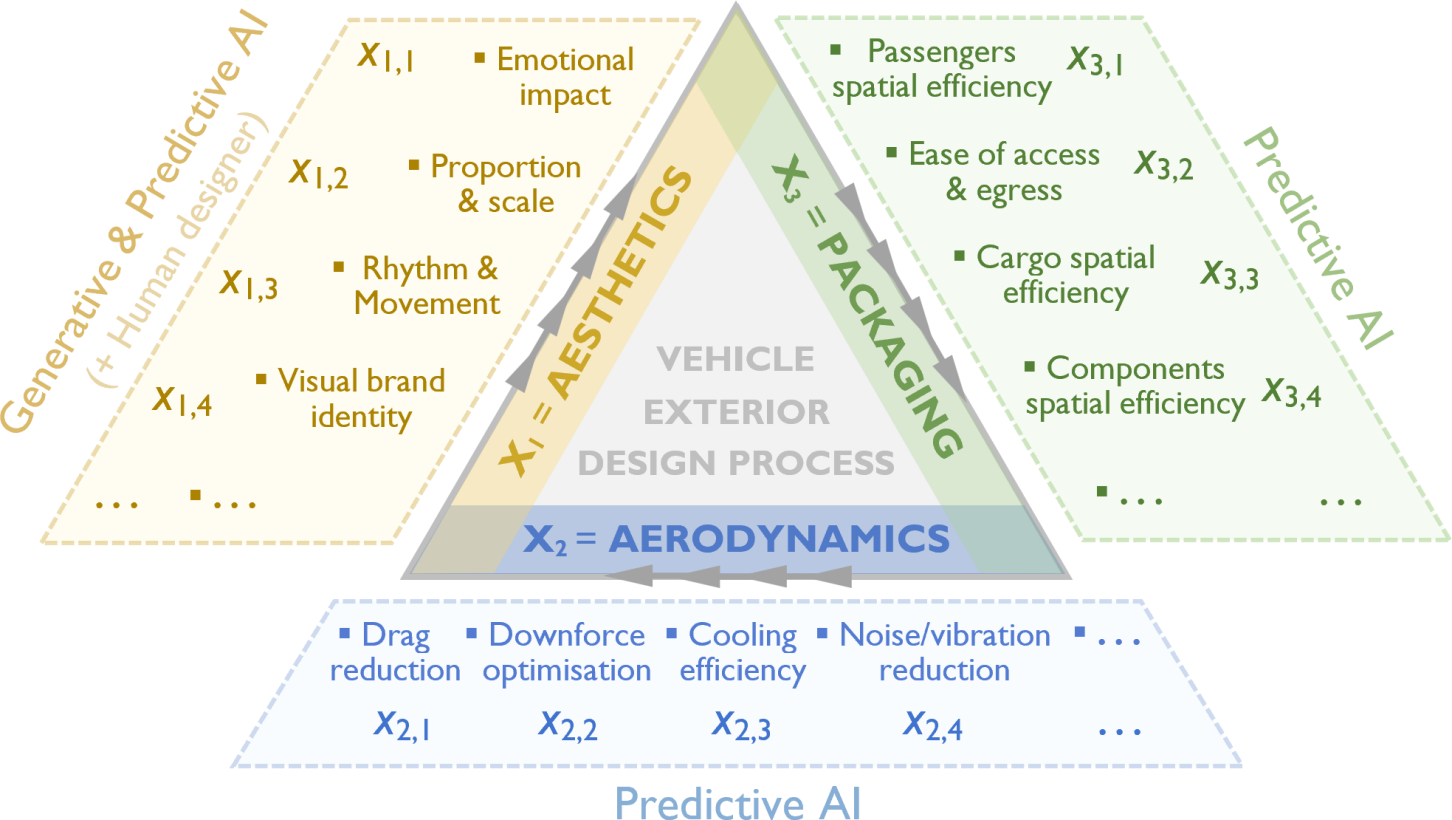
An AI-driven, multi-objective DfX approach to generating and optimizing vehicle exterior design by integrating aesthetics, aerodynamics and packaging.

Figure 25 illustrates an AI-driven, multi-objective approach to vehicle exterior design that integrates aesthetics, aerodynamics and packaging within a structured Design for **X** (Df**X**) methodology. This approach ensures that multiple design criteria are systematically evaluated to achieve a balanced and optimized vehicle exterior.

In this framework, **X** represents key factors influencing design decisions, divided into three primary domains:

(i) *Aesthetics* (**X**₁)—Addressing emotional impact, proportions, rhythm, movement and visual brand identity. This ensures that the vehicle’s form is visually appealing and aligned with the manufacturer’s design language.
(ii) *Aerodynamics* (**X**₂)—Addressing aerodynamic performance aspects such as drag reduction, downforce optimization, cooling efficiency and noise/vibration reduction. These factors are critical in enhancing fuel efficiency, stability and comfort.
(iii) *Packaging* (**X**₃)—Addressing spatial efficiency for passengers, cargo and components, as well as ease of access. Effective packaging ensures practical usability without compromising aerodynamics or aesthetics.

Each of these domains is further broken down into specific criteria (*x_i,j_*), forming a matrix **X** that encapsulates the entire design evaluation process:


(5.1) $$\text{X}=\left[\begin{array}{c} {\text{X}}_{1}\\ {\text{X}}_{2}\\ {\text{X}}_{3} \end{array}\right]=\left[\begin{array}{c} \overset{Aesthetics}{\overbrace{x_{\text{1},\text{1}}\text{​}\;\;\;\;\;\;x_{\text{1},\text{2}}\;\;\;\;\;\;x_{\text{1},\text{3}}\;\;\;\;\;\;\ldots \;\;\;\;\;\;\;\;x_{\text{1},n}}}\\ \overset{Aerodynamics}{\overbrace{x_{\text{2},\text{1}}\text{​}\;\;\;\;\;\;x_{\text{2},\text{2}}\;\;\;\;\;\;x_{\text{2},\text{3}}\;\;\;\;\;\;\;\ldots \;\;\;\;\;\;x_{\text{2},n}}}\\ \overset{Packaging}{\overbrace{x_{\text{3},\text{1}}\text{​}\;\;\;\;\;\;x_{\text{3},\text{2}}\;\;\;\;\;\;x_{\text{3},\text{3}}\;\;\;\;\;\;\ldots \;\;\;\;\;\;\;x_{\text{3},n}}} \end{array}\right]$$

where **X**_1_, **X**_2_ and **X**_3_ are vectors comprising criteria *x_i,j_* associated with aesthetics, aerodynamics and packaging, respectively.

It is important to note that while both aerodynamic and packaging aspects of a design are generally quantifiable, most aesthetic qualities (e.g. emotional impact) are inherently subjective. As a result, appropriate aesthetic evaluation methods—such as data-driven techniques, statistical surveys and expert assessments—must be employed to quantify various aesthetic attributes of the proposed design (for examples of implemented methods, see [139,151–154]).

This structured approach enables designers and engineers to systematically evaluate trade-offs and synergies among aesthetics, aerodynamics and packaging. By utilizing this multi-criterion decision-making matrix, car manufacturers can achieve an optimal balance between design appeal, efficiency and functionality. This approach could be particularly useful in modern automotive development, where aerodynamics significantly impacts electric vehicle efficiency, and packaging constraints are increasingly complex due to battery placement and the thermal management requirements of onboard computational systems, such as advanced driver-assistance systems (ADAS).

## 6. Conclusions

This study has explored the interplay between form and function in vehicle design, tracing the evolution of form–function relationships from early automobile concepts to the emerging AI-driven design methodologies. By classifying formal and functional bioinspiration relevant to early-stage design processes, we have highlighted the influence of biological principles in engineering and industrial design, particularly in the automotive sector. It was highlighted that, while biomimicry has historically played a role in shaping vehicle aesthetics and aerodynamics, its direct impact on functional design has been constrained by the dominance of wheel-based locomotion in ground transportation.

We have examined how streamlining evolved from an aerodynamics-driven principle into a dominant stylistic paradigm and how modern automotive design balances functional efficiency with aesthetic appeal. The historical transition from aerodynamics-led methodologies to styling-led approaches underscores the challenges of integrating scientific principles into consumer-driven markets. The classification of automobile aerodynamic development into four historical phases provides a structured understanding of how design priorities have shifted over time, reflecting technological advancements and changing societal expectations.

The case studies presented in this paper further illustrate the impact of bioinspiration and AI integration in automotive design. Notable examples include the Volkswagen XL1, which drew inspiration from the streamlined body of a dolphin to achieve superior aerodynamic efficiency, and the Bugatti Tourbillon, which emulated the form of the peregrine falcon to minimize air resistance at high speeds. Additionally, the Mercedes-Benz Concept IAA demonstrated how active aerodynamics could dynamically optimize vehicle performance, reflecting natural adaptations seen in birds. These case studies highlight the effectiveness of bioinspired principles in enhancing both form and function while also showcasing the growing role of AI in refining automotive design. By examining these real-world applications, this study reinforces the potential of bioinspiration and intelligent computation to drive future innovations in vehicle development.

A key contribution of this study is the proposal of a fifth historical phase in automotive design, characterized by the integration of AI in styling and engineering. This phase represents a paradigm shift where AI facilitates real-time, concurrent optimization of form and function. By leveraging generative and predictive AI, designers and engineers can collaboratively refine vehicle concepts from the earliest stages of development, ensuring a seamless fusion of aesthetics and performance. The adoption of AI-augmented workflows, such as InStyAO, is expected to streamline the design process, reduce development costs and enhance vehicle efficiency.

Building on this, we introduced an AI-powered, multi-objective framework for vehicle exterior design that seamlessly integrates aesthetics, aerodynamics and packaging within a structured DfX methodology. This approach facilitates a systematic evaluation of multiple design factors to achieve a well-balanced and optimized vehicle exterior. In fact, InStyAO represents a specific application of a broader design methodology, InStylEng, in which multiple functional requirements are addressed alongside aesthetic considerations throughout the design process.

Despite the promise of AI-driven methodologies, challenges remain. The effective implementation of AI in automotive design requires overcoming limitations related to data quality, computational constraints and the interpretability of machine-generated outputs. Additionally, the integration of AI into existing design workflows necessitates interdisciplinary collaboration between designers, engineers and AI specialists. Future research should focus on refining AI-driven design tools, exploring their applicability across different vehicle types, and assessing their long-term impact on sustainability and manufacturing processes.

Looking ahead, the convergence of AI, bioinspiration and computational design has the potential to redefine the automotive industry. As intelligent algorithms continue to evolve, we anticipate a future where vehicle design is no longer dictated by sequential constraints but rather by a dynamic, iterative process that optimally balances aesthetics, performance and sustainability. By formalizing the role of AI in design, this study lays the groundwork for future explorations into how intelligent computation can drive innovation across industrial design disciplines.

Ultimately, this research serves as a foundation for reimagining the future of vehicle design. As AI and bioinspiration continue to shape engineering paradigms, we foresee a new era where automotive design transcends traditional boundaries, embracing an integrated, data-driven approach that harmonizes form and function in unprecedented ways.

## Data Availability

This article has no additional data.

## Footnotes

^1^
Adrian Gaylard (Aerodynamics Research & Development, Jaguar Land Rover), personal email correspondence, 15 January 2025.
